# Evaluation of the molecular inclusion process of β-hexachlorocyclohexane in cyclodextrins†

**DOI:** 10.1039/c9ra04431k

**Published:** 2019-09-02

**Authors:** Anthuan Ferino-Pérez, Juan José Gamboa-Carballo, Ronald Ranguin, Joëlle Levalois-Grützmacher, Yves Bercion, Sarra Gaspard, Ramón Alain Miranda-Quintana, Melvin Arias, Ulises J. Jáuregui-Haza

**Affiliations:** Instituto Superior de Tecnologías y Ciencias Aplicadas (InSTEC), Universidad de La Habana La Habana CP 10600 Cuba ulises.jauregui@intec.edu.do; Department of Chemistry and Applied Biosciences, Laboratory of Inorganic Chemistry, ETH Zürich CH-8093 Switzerland; Laboratoire COVACHIM M2E, Université des Antilles Pointe à Pitre 97157 Guadeloupe France; Department of Chemistry, Université des Antilles Pointe à Pitre 97157 Guadeloupe France; Department of Chemistry, York University Toronto Ontario Canada; Instituto Tecnológico de Santo Domingo, Área de Ciencias Básicas y Ambientales Ave. de los Próceres Santo Domingo Dominican Republic

## Abstract

The present work aimed to study the guest–host complexes of β-hexachlorocyclohexane (β-HCH), a pesticide with high environmental stability that can cause severe health problems, with the most common cyclodextrins (α-, β-, and γ-CDs). The formation reactions of these molecular inclusion complexes were addressed in this research. The multiple minima hypersurface methodology, quantum calculations based on density functional theory and a topological exploration of the electron density based on the quantum theory of atoms in molecules approach were used to characterize the interaction spaces of the pollutant with the three CDs. Additionally, charge distribution, charge transfer and dual descriptor analyses were employed to elucidate the driving forces involved in the formation of these molecular inclusion complexes. Three types of fundamental interactions were observed: total occlusion, partial occlusion and external interaction (non-occlusion). Finally, experiments were performed to confirm the formation of the studied complexes. The most stable complexes were obtained when γ-CD was the host molecule. The interactions between the pesticide and CDs have fundamentally dispersive natures, as was confirmed experimentally by spectroscopic results. All the obtained results suggest the possibility of using CDs for the purification and treatment of water polluted with β-HCH.

## Abstract 2

The present work aimed to study the guest–host complexes of β-hexachlorocyclohexane (β-HCH), a pesticide with high environmental stability that can cause severe health problems, with the most common cyclodextrins (α-, β-, and γ-CDs).

## Graphical abstract

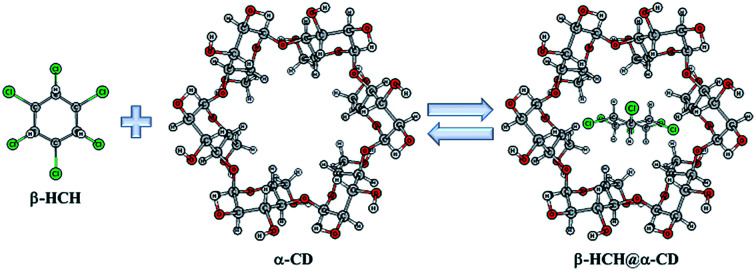

## Introduction

HCH is a term that defines, in a collective manner, the isomers of hexachlorocyclohexane, a monocyclic, chlorinated, and saturated hydrocarbon with the chemical formula C_6_H_6_Cl_6_. Technical HCH is synthesized through the photochemical chlorination of benzene to produce a mixture of isomers that differ in the axial/equatorial distributions of the chlorine atoms in the cyclohexanes; these are denoted with the Greek letters α, β, γ, δ, ε, η, and θ. Among these isomers, γ-HCH (lindane, CAS 58-89-9) is the only one with insecticidal properties. However, its commercial mixture contains up to 87% of the other isomers, mainly α-, β-, and δ-HCH, even though all of these are toxic and carcinogenic.^1^

Nearly 600 000 tons of technical HCH compounds were used in several countries between the decades of 1940 and 1990 with the aim of controlling a wide range of agricultural and health plagues.^2,3^ Places heavily polluted with HCH have been reported all over the world.^4–7^ Due to its persistence, tendency to bioaccumulate, carcinogenic properties, and ability to act as an endocrine disruptor, the use of lindane is banned in at least 52 countries.

**Fig. 1:**
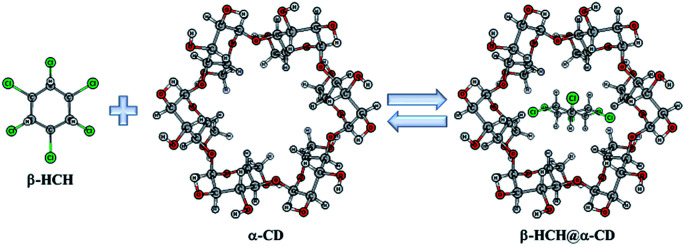
Reaction of β-hexachlorocyclohexane (β-HCH) with cyclodextrins (CDs). α-CD is used as example of a host molecule.

The HCH isomers can be detected in all the compartments of the environment, including water, soil, air, and biota. As a result of the water cycle, HCH in the land polluted with HCH is transferred progressively to aquatic ecosystems. Its high adsorption coefficient in soil organic carbon (log *K*_oc_ ≈ 3.57) demonstrates its great affinity for topsoils rich in organic matter. While the α and γ isomers are the most widely dispersed in the environment, β-HCH is the one with the greatest tendency to bioaccumulate; it can be transported by air and has the greatest stability.^8^ In long-term studies about the persistence of the HCH isomers, β-HCH was found to be the most persistent due to its structure (all Cl atoms are equatorial, as shown in Fig. 1) and its higher boiling temperature, with 44% remainder after 15 years.^9^ Also, β-HCH's relatively high octanol/air partition coefficient (*K*_oa_ = 5.1 × 10^8^)^10^ promotes accumulation from the air in environmental organic phases. This is probably the reason for the differences in the transport mechanisms between β-HCH and the other isomers in the environment.

Technically, HCH can enter the food chain, bioaccumulate, and cause biomagnification, especially in fish, birds, and other animals that feed on sediments, such as crabs and turtles;^7,8^ it even affects their reproduction. Notably, β-HCH is the isomer with the greatest tendency to bioaccumulate.^11^ On the basis of wide analysis of the physicochemical property data of α-HCH, γ-HCH, and β-HCH, it is possible to state that the greater tendency of the latter to bioaccumulate is due more to its greater resistance to metabolic conversion than to its greater solubility in lipids.^10^ Furthermore, the predominant formation of significant amounts of β-HCH from other isomers has been demonstrated. This results in an increment of its stability and a decreased bioremediation rhythm, causing an increase of its environmental persistence.^12^ It has been also established that β-HCH is neurotoxic, hepatotoxic, causes fertility problems, has immunosuppressive effects and acts as an endocrine disruptor.^13^

As a result of all the previously discussed aspects, in 2009, β-HCH (CAS 319-85-7) was included in the list of persistent organic pollutants (POPs) by the Stockholm Convention.^14^ This prompted a search for new decontamination strategies focused on this pollutant. For example, in recent years, several studies of different degradation methods for HCH isomers have been carried out. These methods range from advanced oxidation^15^ and bioremediation treatments^9,16,17^ to the use of activated carbon for the treatment of polluted water.^18–20^ In spite of these efforts, it is still necessary to increase the efficiency of the separation methods used; this has promoted the search for new alternatives, such as the formation of host-guest complexes with cyclodextrins.

Cyclodextrins (CDs) are a family of cyclic oligosaccharides consisting of a number of α-d-glucopyranose subunits linked by (1 → 4) glycosidic bonds. The more common CDs, called α-, β- and γ-CDs, are formed, respectively, of six, seven and eight glucopyranose units.^21^ Table S1 in the ESI† shows the characteristic dimensions of these cyclodextrins. The remarkable encapsulation capacity of CDs results in a type of host–guest relationship^22,23^ that can modify and/or improve the physicochemical and biological properties of the guest molecule.^21^ This has led to applications in agriculture and the environment.^24,25^ Particularly interesting for our present purposes, we should highlight their use in innovative alternatives to remove pollutants from waters and soils based on the formation of non-soluble inclusion complexes in water and in most organic solvents.^26,27^ In 2016, Rana *et al.* successfully used CDs for the removal of kepone from aqueous solutions and for the modification of adsorbent systems, such as activated carbon filters, considerably increasing their efficiency.^26^ β-HCH is an organochlorine pesticide as well; this suggests that it is possible to use CDs for its removal.

The mathematical modeling of the interactions of pollutants/decontaminant agents through the application of computational chemistry methods is a tool that has been recently used to facilitate the management of hydric resources in the environment. These methods allow economy of material resources while optimizing the time and safety of researchers. Particularly, the molecular inclusion complexes formed between the CDs and different molecules have been studied by several computational methods, including molecular docking,^28,29^ molecular dynamics,^30,31^ density functional theory (DFT) calculations,^31–33^ and natural bonding orbitals and quantum theory of atoms in molecules approaches.^34–36^ However, in these studies, the authors directly set the molecule of interest in contact with the interior of the CD cavity in certain configurations, based largely on chemical intuition. Also, generally, only β-CD is considered as a host molecule; this ignores the possibility of better encapsulation capacities of other CDs, especially γ-CD. In this work, a more general approach was used based on random exploration of the configurational spaces of the host–guest complexes of β-HCH in each of the three naturally occurring CDs (α-, β-, and γ-CD).

The aim of this work is to theoretically characterize the interactions between β-HCH and the α-, β- and γ-CDs. This permits analysis of the formation and stability of the possible molecular inclusion complexes of β-HCH@CDs (Fig. 1). The precipitation of nanoaggregate β-HCH@CDs from aqueous solutions was experimentally demonstrated. The obtained results shed light on their use as an alternative for the removal of this pesticide from polluted waters.

## Theoretical and experimental methods

### System under study

**Fig. 2:**
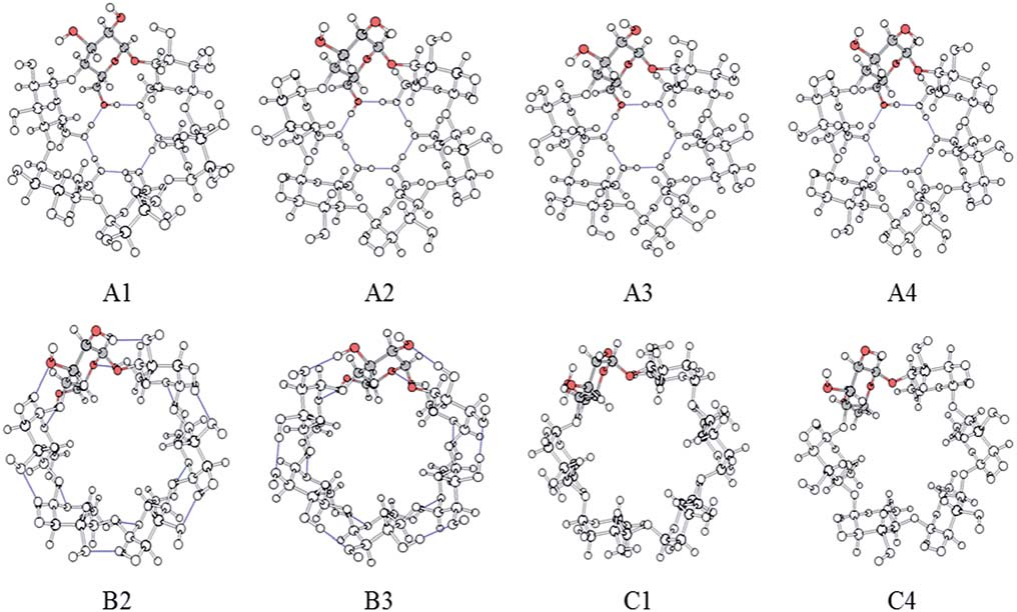
Symmetrical conformers of CDs. Only α-CD is presented, and a glucopyranose unit is highlighted in each conformer.

Due to the size of the pollutant, only complexes with a stoichiometry of 1 : 1 were considered. For more rigorous characterization of the interactions between the CDs and the pesticide (β-HCH), different conformers of the CDs were studied. A group of twenty-four symmetric conformers was used, with eight for each CD. These conformers were previously characterized in recent work^37^ and differ in the orientation patterns of their intramolecular hydrogen bonds. The conformers were classified into three groups: A, B, and C. The labels in the classification of the conformers refer to the orientation of the hydrogen bonds in clockwise and anti-clockwise ways.^37^Fig. 2 shows the eight conformers for α-CD.

### Multiple minima hypersurface calculations

The first step of this work was to carry out an exploration of the interaction space of β-HCH@CD using the Multiple Minima Hypersurface (MMH) methodology.^38^ Although the Gibbs free energy of association (Δ*G*_assoc_) is the thermodynamic criterion used to define the spontaneity of a process, here, the association energy (Δ*E*_assoc_) was preferred. This criterion has been widely used in the past^39–42^ because it is simpler from a computational point of view; also, it avoids the necessity of calculating the association entropies. Δ*E*_assoc_ is defined as:

1 Δ*E*_assoc_ = *E*_supermolecule_ − *E*_reference_

*E*_supermolecule_ is the energy of the molecular complex formed between the CD and β-HCH, while *E*_reference_ is the sum of the energies of each independent molecule. A favorable thermodynamic association implies that the supermolecule will be more stable than the isolated molecules; namely, a greater absolute value of Δ*E*_assoc_ corresponds to more energetically favorable associations.

The MMH methodology usually works with semiempirical methods^39–44^ for the evaluation of the energies while using statistical mechanics to obtain thermodynamic properties related to the molecular association.^38^ The main procedure of this approach constructs several random non-redundant molecular geometries, starting from the independently optimized structures of the interacting molecules, in order to explore the configuration space of the formed complexes. It is important to note that the term “association energy” represents the thermodynamic association energy calculated through the statistically weighted sum (*i.e.* partition function) of the representative “supermolecule” states.^45^ A Boltzmann distribution is used to calculate the thermally averaged state of the typical macroscopic system at room temperature (298.15 K).

The standard MMH procedure places the CD in the center of a cubic box and then places the β-HCH molecules on random configurations inside this box using Granada software.^46^ As previously stated in the System under study section, only 1 : 1 complexes were considered; in consequence, only one of each reactant was used to explore the potential energy surface of the system. In this work, solvent molecules were not explicitly used.

The semiempirical Hamiltonian PM6-D3H4X^47–50^ was used for the optimization of the 200 geometries of each system and the calculation of their respective energies using the software MOPAC2016.^51^ PM6 is a modern semiempirical Hamiltonian that improves the description of the H-bonds with respect to previous semiempirical methods.^47^ For a more precise description of the system, two corrections were introduced: the first one, D3H4,^49,50^ integrated advanced corrections to the dispersive and H-bond interactions; the second, X,^48,50^ rectifies the failure present at PM6 when describing the interactions of halogens (Cl, Br, I) with more electronegative atoms, such as O and N. This is because the basis functions used in PM6 do not accurately describe the anisotropy of the electronic density on the halogen atoms (σ-holes), which is responsible for the contribution to the electrostatic attraction in the bonds of the halogens with the O and N atoms.

### Re-optimization of distinctive structures

Afterward, the representative structures (*i.e.*, the most stable minima of the CD-pollutant complexes) were re-optimized using DFT.^52,53^ For this re-optimization, a hybrid functional that uses the meta-generalized gradient approximation M06-2X^54,55^ was used, along with the 6-31G(d,p) basis set. It has been reported that this combination correctly describes the interactions found in non-covalently bonded dimers,^55^ particularly in the case of the van der Waals interactions present in the studied systems. DFT-D3 dispersive corrections^56^ were applied to all DFT calculations; thus, hereafter, the D3 part in the M06-2X-D3 method will be omitted for simplicity and readability. The solvent effects were taken into account using the solvent implicit model SMD^57^ for all DFT calculations. The method of Gamboa *et al.* was used to mitigate the basis set superposition error (BSSE),^58^ which enables solvent effects to be taken into account at the same time. All the calculations were performed using the Gaussian 09 software package.^59^

### Study of chemical properties

Following the geometrical re-optimization of the selected minimum energy structures, single-point calculations were performed using the UM062X/6-311G(2df,2pd) theory level, maintaining SMD as the implicit solvent model. Analogous calculations were performed for the corresponding cationic and anionic species. This allowed us to examine several properties of interest and their implications in the formation processes of the complexes, such as charge distribution and transfer. Note that because this requires performing calculations on anions, the use of diffuse functions in the basis set was purposely avoided, as this is usually problematic for metastable anions.^13,60–64^

The charge distribution study was performed by means of population analysis of the atomic charges using Hirshfeld population.^65^ This method is based on deformation density partition and provides results that are qualitatively consistent with general chemical concepts such as atomic and group electrophilicity.^66,67^ Although calculating Hirshfeld charges requires integration in real space, due to the smooth integrand, sophisticated DFT grid-based integration schemes can be directly used; thus, Hirshfeld population is a highly efficient method and has a wide application field.^68^

For the analysis of the local charge transfer tendencies, the dual descriptor^69^ (Δ*f*(*r*)) was calculated. This descriptor plays an important role in conceptual DFT^70–76^ and has been widely used in the description of charge-transfer driven reactions.^77–79^ Two types of analysis are typically used: by means of the standard local descriptor (*i.e.*, using the tridimensional plot of the dual descriptor) and its atom-condensed form.^80,81^ The first procedure is based on the typical (finite-difference) definition of the dual descriptor:^75^

2 Δ*f*(*r*) = *f*^+^(*r*) − *f*^−^(*r*)

3 Δ*f*(*r*) = *ρ*_*N*+1_(*r*) − 2*ρ*_*N*_(*r*) + *ρ*_*N*−1_(*r*)

Here, *f*^+^(*r*) and *f*^−^(*r*) are the Fukui functions from above and from below,^82^*ρ* is the electron density and *N* is the number of electrons in the molecule. Unlike the Fukui function, Δ*f* is sufficient to describe both the nucleophilic and the electrophilic behavior of the molecule: if Δ*f* > 0, then the site is favorable for nucleophilic attack, whereas if Δ*f* < 0, the site is favorable for electrophilic attack. The variant based on the atomic charges works with the condensed Fukui functions; in consequence, the condensed dual descriptor allows us to understand the changes in reactivity at the atomic level. The Hirshfeld population analysis was also used in this case. The expressions analogous to eqn (2) and (3) are:

4 Δ*f*_A_ = *f*^+^_A_ − *f*^−^_A_

5 Δ*f*_A_ = 2*q*^A^_*N*_ − *q*^A^_*N*+1_ − *q*^A^_*N*−1_

In this case, *f*^+^_A_ and *f*^−^_A_ are the condensed Fukui functions, Δ*f*_A_ is the condensed dual descriptor, and *q* is the atomic charge; note that we have used the “response of molecular fragments” approach to condense the reactivity indices.^83,84^

The charge transfer processes were investigated by subtracting the electron density of β-HCH and the corresponding cyclodextrins in the standalone state from the electron density of the formed complexes.

### Topological analysis of the electron density

To obtain a better description of the interactions present between the CDs and the pesticide, the electronic densities of the neutral complexes were re-optimized using the 6-311++G(2df, 2pd) basis set. The representative structures of the β-HCH@CD complexes were studied using the quantum theory of atoms in molecules (QTAIM);^85,86^ this enables the analysis of the nature of the interactions based on Nakanishi's criteria,^87,88^ which is one of the main goals of this investigation. This analysis was performed using the Multiwfn 3.3.6 software package.^89^

### Experimental confirmation of the formation of guest–host complexes

#### Preparation of β-HCH@cyclodextrin complexes

Experimental confirmation of the formation of the β-HCH@CDs complexes was obtained. CDs of analytical grade (>98%) were purchased from Merck and Fluka.^90^ β-HCH was purchased from Sigma Aldrich Chemical Company (St. Louis).

Solutions of α, β, and γ-CD, 0.01 mmol·L^−1^ each, were prepared in deionized water; also, a solution of β-HCH in a stoichiometric amount was prepared by the previous dilution of pesticide in a minimum amount of methanol. After solubilization in their respective solvents, 10 mL of the host and guest compound solutions were mixed together, and the appearance of a white precipitate, indicative of the formation of inclusion β-HCH@CD complexes, instantaneously occurred. The supernatant was decanted and the solid was washed with a mixture of methanol–water (1 : 1) solution. The obtained precipitate was then dried under vacuum at room temperature in a desiccator over silica gel.

#### β-HCH quantification

Quantification of β-HCH in aqueous solution was carried out with a liquid chromatograph (LC) coupled to a mass spectrophotometer (AGILENT LC/MS 1100 series system). The LC separation was performed using a C8 column (2.1 × 150 mm, Eclipse X08-C8) at 80 °C using the following gradient: 0 to 6 min 55% ACN in water, one minute more at 100%. Ionization of β-HCH was achieved by electrospray (API-ES) in negative ion mode. The final parameters of the nebulizer chamber were drying gas flow of 12 L min^−1^ at 350 °C; the pressure of the atomizer: 35 psi; capillary tension: 4000 V; collision energy: 50 eV. The product-ion mass spectra of β-HCH are *m*/*z* 507 and 509.

#### Spectroscopic characterization techniques

Raman scattering spectra of the CDs (α, β, and γ-CD), β-HCH, and samples of the different formed precipitates were measured using a Horiba Scientific LabRAM HR Evolution microscope system with a laser beam line Ar^+^ ion of 514.5 nm. Accumulation times were 20 s. Fourier transform infrared (FTIR) spectra were collected using a PerkinElmer Spectrum One FTIR Infrared Spectrometer with an ATR sampling accessory (diamond/ZnSe composite crystal) using a spectral resolution of 4 cm^−1^ for 8 scans.

#### Morphological characterization of the precipitates

TEM analysis was performed using a FEI Tecnaï F 20 XTwin microscope with a field emission gun running at 200 kV.

## Results and discussion

### MMH calculations. Formation of the β-HCH@CD inclusion complexes

**Fig. 3:**
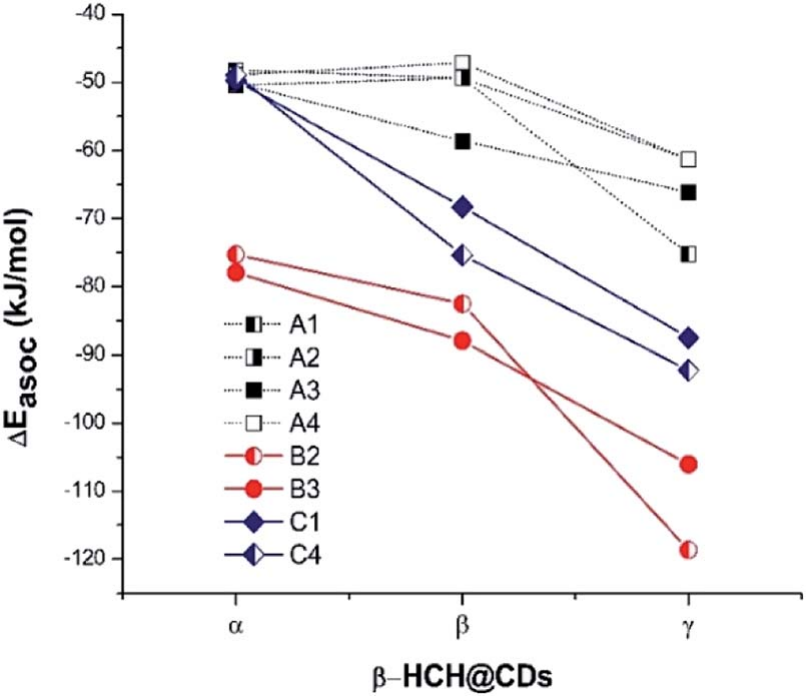
Mean association energies (calculated by the MMH methodology) of β-HCH with the conformers of the studied CDs.

In each of the 24 studied systems, 200 random structures were generated and optimized in order to explore the configuration spaces of the interactions of β-HCH and the three CDs. Fig. 3 shows the behavior of the mean association energies obtained for the described systems at 298.15 K. Table S2 in the ESI† shows all the thermodynamic magnitudes calculated through the MMH procedure.

From Fig. 3, it is possible to observe that the complexes β-HCH@β-CD have similar energies with respect to the complexes formed with α-CD for the conformers A1, A2, and A4. These conformers should not predominate, in accordance with the experimental and theoretical data reported in the literature.^37,91,92^

In contrast, Fig. 3 shows progressive stabilization of the conformers A3, B2, B3, C1, and C4. It is clear that the stabilization of the system β-HCH@CD is favored when γ-CD is the host molecule, given the higher association energies. In the particular cases of the conformers A1, A2, and A4, the order of the forces of association is β-HCH@α-CD ≈ β-HCH@β-CD < β-HCH@γ-CD. However, in general, the order of the association forces for the conformers is β-HCH@α-CD < β-HCH@β-CD < β-HCH@γ-CD.

**Fig. 4:**
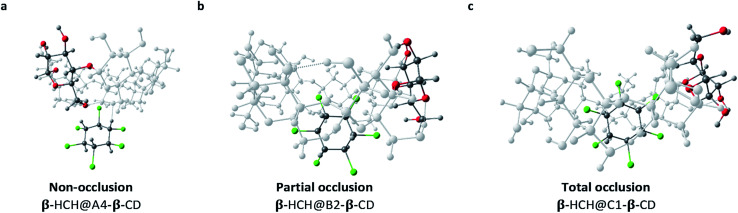
Classification according to the degree of occlusion of the interactions of β-HCH@CD. In all cases, β-CD is the host molecule.

The differences between the degrees of occlusion presented by the formed complexes allow us to classify them in three groups (Fig. 4): external interaction without occlusion (non-occlusion) (Fig. 4a), partial occlusion (Fig. 4b), and total occlusion (Fig. 4c).

Table 1 illustrates the predominant degrees of occlusion for each of the conformers of the CDs. As can be observed when α-CD and β-CD are the host molecules, partial and non-occlusion predominate, which is due to the small sizes of the cavities of these CDs. Meanwhile, when γ-CD is the host molecule, total occlusion prevails, accounting for the better encapsulation capacity of this CD due to its larger cavity size.

**Table 1:** Distribution of the degrees of occlusion for each conformer of α-CD, β-CD, and γ-CD obtained by the MMH methodology

Host CD conformer	% Total occlusion	% Partial occlusion	% Non-occlusion
A1-α-CD	0.0	0.0	99.9
A2-α-CD	0.0	0.5	99.4
A3-α-CD	0.0	0.00	99.9
A4-α-CD	0.0	0.00	99.9
B2-α-CD	0.0	99.7	0.2
B3-α-CD	0.0	99.6	0.3
C1-α-CD	0.0	94.3	5.7
C4-α-CD	0.0	96.7	3.2
A1-β-CD	0.0	9.3	90.6
A2-β-CD	0.0	1.7	98.2
A3-β-CD	0.5	0.7	98.7
A4-β-CD	5.6	11.5	82.6
B2-β-CD	0.3	99.6	0.0
B3-β-CD	0.0	99.7	0.2
C1-β-CD	46.7	50.7	2.6
C4-β-CD	0.00	46.8	53.2
A1-γ-CD	99.9	0.0	0.0
A2-γ-CD	94.0	0.0	5.9
A3-γ-CD	98.3	0.0	1.6
A4-γ-CD	99.6	0.0	0.3
B2-γ-CD	100.0	0.0	0.0
B3-γ-CD	28.7	70.2	0.6
C1-γ-CD	94.6	0.9	4.5
C4-γ-CD	92.4	0.0	7.5

Moreover, the MMH/PM6-D3H4X calculations suggest the existence of two main interaction types: the formation of H-bonds between the axial hydrogens of β-HCH and the oxygens of the primary hydroxyl of the CD, and dispersive interactions of the chlorine atoms with the interior of the CD cavity. The association energies and the obtained geometries suggest that the complexes with greater stability should be those where more chlorine atoms are inside the hydrophobic interior of the CD (systems with a greater degree of occlusion).

From these results (complexes with great thermodynamic stability and a considerable degree of occlusion), it could be expected that for β-HCH, the behavior should be similar to that reported by Rana *et al.*^26^ for the system chlordecone@CDs, which experimentally confirms the preference for inclusion complexes when γ-CD is the host molecule.

The MMH procedure indicates that the conformers B2, B3, C1, and C4 are the most stable complexes. Of these conformers, a total of 15 representative structures of inclusion complexes of β-HCH@CDs were selected for posterior refinement. In all cases, the global minima and additional structures of interest for the presented geometry were selected. This selection was made while always taking into account that these structures have a population greater than 10% according to the Boltzmann distribution.

### Refinement of the representative structures using DFT M06-2X

**Fig. 5:**
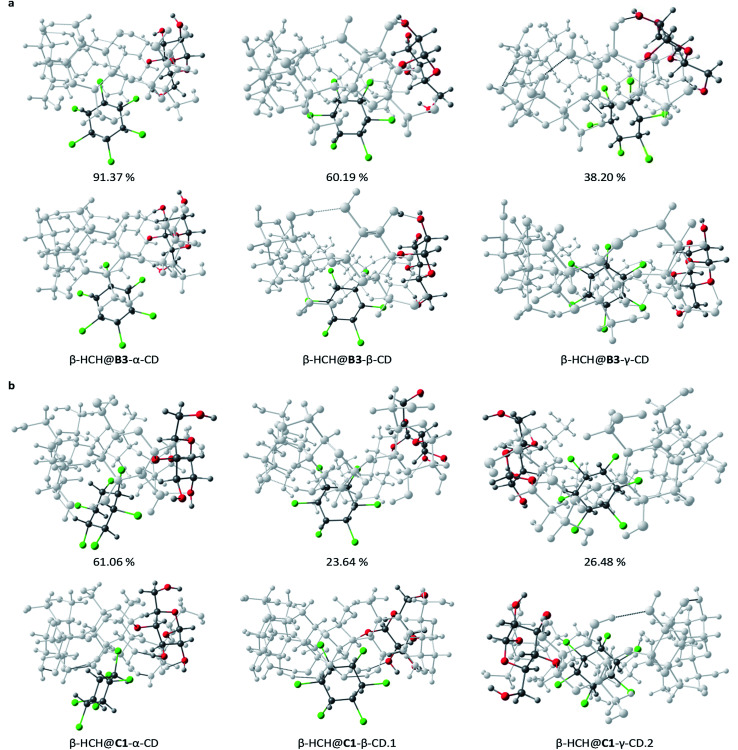
Distinctive structures for the most stable CD conformers, B3 (a) and C1 (b), studied at different theory levels: PM6-D3H4X (top) and DFT M06-2X (bottom).

The 15 selected complexes were re-optimized using the M06-2X/6-31G(d,p) scheme. This allows us to describe the parameters of interest (*i.e.* molecular geometry and stability) with greater precision. The association energies, obtained from the re-optimization, are shown in Table 2. Only small variations of the geometries calculated through DFT from the ones obtained by MMH were observed. The most abrupt changes occur in the complex β-HCH@B3-γ-CD, which after the re-optimization changed from partial to total occlusion of the pollutant inside the CD cavity (Fig. 5a), stabilizing the complex.

**Table 2:** Values of the association energies for selected structures using DFT M06-2X

Complex^a^	Degree of occlusion of the cavity	Δ*E*_assoc_ M06-2X [kJ mol^−1^]	Corrected^b^ Δ*E*_assoc_ M06-2X [kJ mol^−1^]	% BSSE^b^
β-HCH@B2-α-CD	Partial occlusion	−59.66	−35.08	41.19
β-HCH@B3-α-CD	Partial occlusion	−51.73	−17.33	66.50
β-HCH@C1-α-CD	Partial occlusion	−87.89	−47.74	45.68
β-HCH@C4-α-CD	Partial occlusion	−63.10	−42.31	32.95
β-HCH@B2-β-CD	Partial occlusion	−81.05	−49.16	39.35
β-HCH@B3-β-CD	Partial occlusion	−82.45	−49.11	40.44
β-HCH@C1-β-CD.1	Partial occlusion	−99.16	−58.70	40.80
β-HCH@C1-β-CD.2	Partial occlusion	−94.07	−51.98	44.74
β-HCH@C4-β-CD.1	Partial occlusion	−60.15	−41.53	30.96
β-HCH@C4-β-CD.2	Partial occlusion	−82.21	−42.62	48.16
β-HCH@B2-γ-CD	Total occlusion	−72.43	−34.49	52.38
β-HCH@B3-γ-CD	Total occlusion	−94.41	−62.97	33.30
β-HCH@C1-γ-CD.1	Total occlusion	−109.55	−67.51	38.37
β-HCH@C1-γ-CD.2	Total occlusion	−108.03	−79.57	26.34
β-HCH@C4-γ-CD	Total occlusion	−95.66	−59.44	37.86

a In the case that more than one structure is presented, (1) denotes the global MMH minimum and (2) denotes another structure of interest.

b Energies are reported after and before BSSE correction using the method developed by Gamboa *et al.*^58^ was applied.

Table 2 also shows the values of the association energies after correction of the BSSE using the method proposed by Gamboa *et al.*^58^ As observed, the stabilization of the complexes due to the BSSE is significant, where the amount of corrected energy is in the range of 20 to 40 kJ mol^−1^ in almost all cases. The importance of correcting the BSSE is such that in several cases, changes in the order of stability even within the different PM6-D3H4X minima were observed, as in the cases of β-HCH@C1-γ-CD.1 and β-HCH@C1-γ-CD.2. All the necessary data to perform the BSSE correction using the thermodynamic cycle are presented in Tables S3–S5 of the ESI.†

The values in Table 2 reflect that the conformers C1 and C4 are the most stable, which can be explained by taking into account that the complexes formed with these conformers show greater degrees of occlusion. Furthermore, they present secondary hydroxyl groups oriented toward the solution; this endows them with additional stability for the possibility of electrostatic interactions and polarization with the solvent. The results obtained for the molecular inclusion complexes formed with β-CD are in agreement with other DFT studies.^30,32,35,36^ As in the present work, predominant partial occlusion is observed when β-CD is the host molecule.

The obtained results indicate that the most stable inclusion complexes are formed when γ-CD is the host molecule for the conformers B3, C1, and C4. For the conformer B2, the complex β-HCH@β-CD is the most stable. From this fact, it can be concluded that in general, the most stable complexes are those with the greatest degrees of occlusion. Fig. S1 of the ESI† presents the optimized geometries for the 15 complexes, while Fig. 5 shows a representation of the most stable complexes for each type of CD conformer.

### Study of the properties of the complexes

**Fig. 6:**
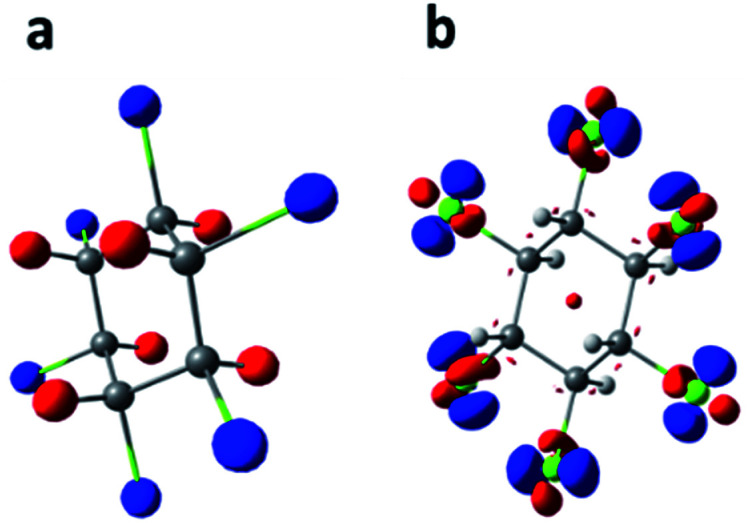
Electrostatic potentials of β-HCH from nuclear charges (a, isosurface contour value 0.1 a.u.) and dual descriptor (b, isosurface contour value 0.5 × 10^−2^ a.u.). Blue represents negative values and red represents positive values.

Afterward, recalculations of the electronic densities of β-HCH were performed for the complexes formed with conformers B2 and C1 and their respective anions and cations. For these recalculations, the UM06-2X/6-311G(2df, 2pd) scheme was used. These conformers were selected because they are representative of the B and C types, respectively. An analysis of the changes in several properties of the pollutant, such as charge distribution and reactivity, was performed. Fig. 6a shows the electrostatic potential from the atomic charges obtained after the Hirshfeld population analysis of β-HCH. Fig. 6b displays the dual descriptor for this pesticide. As can be seen, the chlorine atoms have a negative charge and are the most reactive, as expected.

**Fig. 7:**
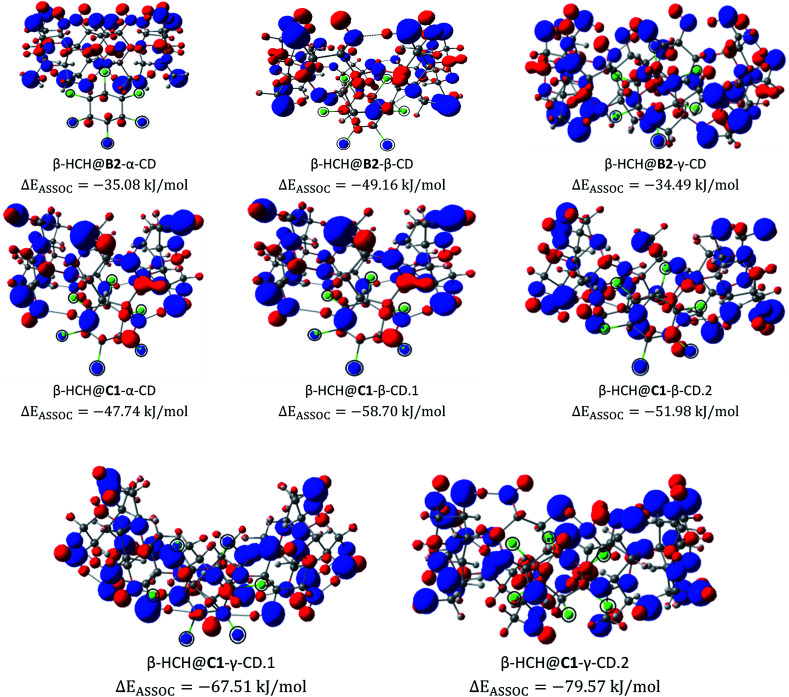
Electrostatic potentials from nuclear charges for the studied complexes of the conformers B2 and C1. The chlorine atoms are encircled for clarity (isosurface contour value 0.1 a.u.).

The electrostatic potentials (ESP) from nuclear charges of the studied complexes are presented in Fig. 7. When compared with the ESP from the nuclear charges of β-HCH before the formation of the complex, it can be seen that the interaction of the chlorine atoms with the inner cavity of the cyclodextrins is characterized by a transfer of charge density from these chlorine atoms to the cyclodextrins. These interactions induce a redistribution of the atomic charges and, therefore, a decrease of the charges of the occluded chlorines. Also, when the stabilities of different complexes of the same CD are compared, it can be noted that the stability increases with the number of Cl atoms participating in the donation process. This can be seen in the complexes β-HCH@C1-β-CD.1 and β-HCH@C1-β-CD.2 and in greater proportion in β-HCH@C1-γ-CD.1 and β-HCH@C1-γ-CD.2. Therefore, it is possible to conclude that these interactions play a key role in the stabilization of the complexes.

**Fig. 8:**
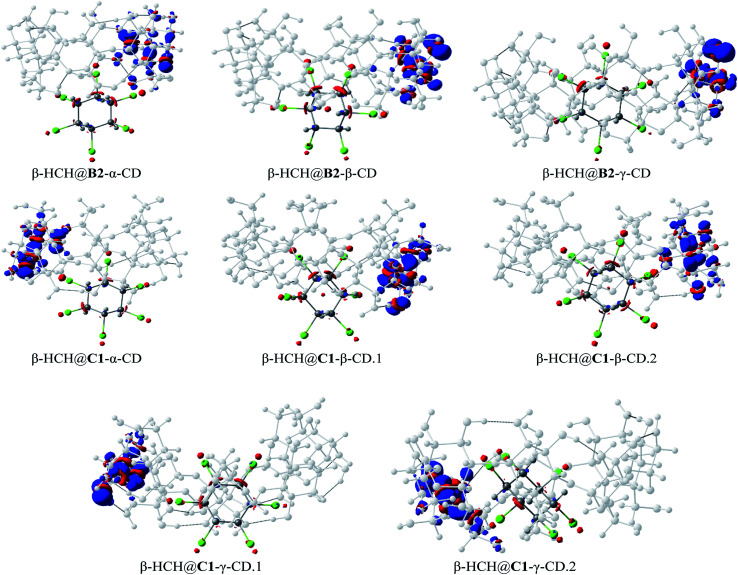
The dual descriptors of the studied complexes for the conformers B2 and C1 (isosurface contour value 0.5 × 10^−2^ a.u.).

Fig. 8 shows the dual descriptors. An important decrease in the reactivity of the atoms of the pesticide can be observed in comparison with Fig. 6b. Specifically, the sites of electrophilic attack on the chlorine atoms present in β-HCH before the formation of the complexes are no longer practically available. The condensed dual descriptors for all the complexes agree with these results and are available in Table S6 of the ESI.† This “protection” of chlorine atoms from electrophilic attack by occlusion inside the CD cavity is in accordance with the donation of electrons during the formation of the complexes, as previously discussed. As can be observed in Fig. 8, in all cases, no matter the degree of occlusion, a glucopyranose unit is the most reactive part of the complex.

Additionally, the evaluation of the charge transfer process can be seen in Fig. S2 of the ESI.† If we closely analyze the CD atoms which gained charge during the occlusion process (Fig. S2†), it can be noted that the charge is not transferred through the glucopyranose unit. This implies that the process of formation of the complexes should not affect the behavior of the exterior surface of the CD molecules.

**Fig. 9:**
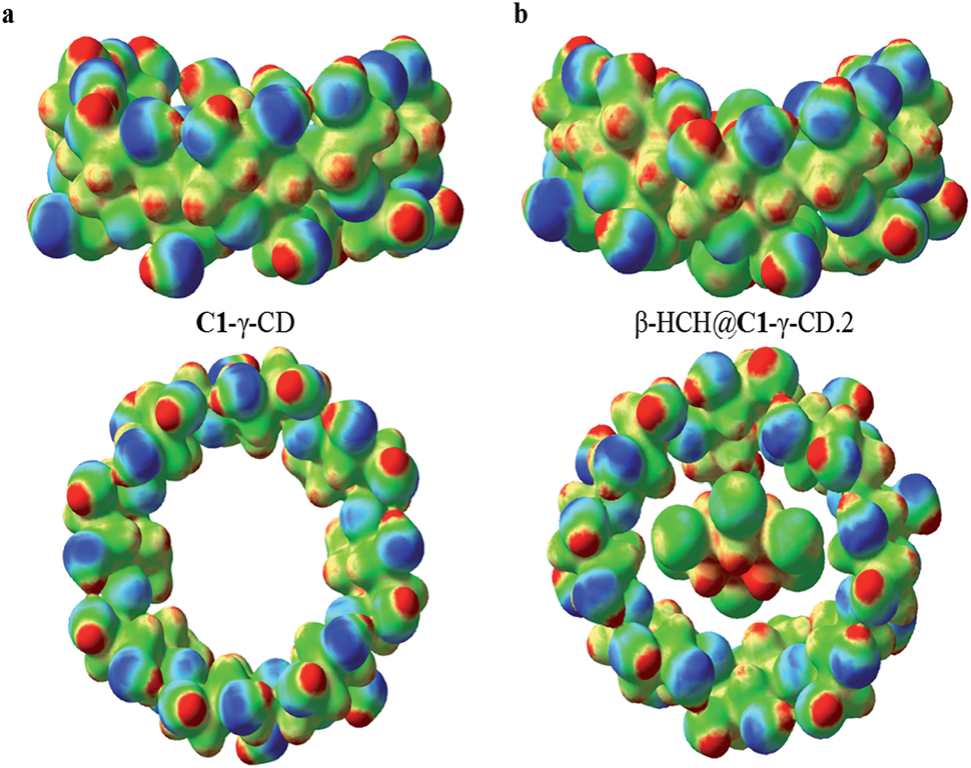
Maps of the total electrostatic potential for (a) C1-γ-CD and (b) β-HCH@C1-γ-CD.2. Electron density isosurface contour value 0.2 a.u.; the total ESP values range from −5.5 × 10^−2^ a.u. (blue) to 0.2 a.u. (red).

In order to corroborate this last observation, the total electrostatic potentials for C1-γ-CD and the complex β-HCH@C1-γ-CD.2 (the more stable of the studied complexes) were mapped. The results of this mapping of the total ESP are shown in Fig. 9. Here, the isosurface contour value 0.2 a.u. was chosen to account for the reactive surface of the studied molecules. The total ESP values range from −5.5 × 10^−2^ a.u. (blue) to 0.2 a.u. (red); light blue, green and yellow respectively represent the progressive increment of the total ESP between these two limits. As can be seen, there are no abrupt changes in the electrostatic potential of the external surface of the CD, which agrees with the results of the charge transfer analysis. However, it is important to note that the interaction with β-HCH causes a disruption in the symmetry of the total electrostatic potential; this may be a factor that affects the solubility of the studied complexes.

These results suggest that one way to increase the stability of these complexes and favor their formation is introducing electron-withdrawing substituents into the glucopyranose units, which should contribute to the process of electron transfer from the pesticide to the inner cavities of the cyclodextrins.

### QTAIM analysis

For a more rigorous characterization of the interactions in β-HCH@CDs complexes, a topological analysis of the electron density (QTAIM) was performed. The application of QTAIM analysis allowed us to characterize the topology of the electron density (*ρ*) and its Laplacian (∇^2^*ρ*) at the bond critical points (BCPs) and therefore to describe the molecular interactions and to classify them according to generally accepted criteria. In this work, Nakanishi's criteria^87,88^ were used for this purpose. Furthermore, other ρ-dependent functions, such as the total energy density (*H*_BCP_), the potential-kinetic energy density ratio (*V*_BCP_/*G*_BCP_), and the ellipticity of the electron density (*ε*), were used.

From the mentioned parameters, it was possible to confirm the predominance of dispersive van der Waals interactions (vdW) between the pollutant atoms and those of the interior of the CD cavity. Even in the case of the complexes where occlusion does not occur to a great extent (mainly β-HCH@α-CD), a great number of non-covalent interactions with the atoms of the narrower ring of the CD were found (with a minimum of 17 interactions). Table 3 shows the QTAIM analysis of the representative interactions of the β-HCH@C4-β-CD.2 complex. The totality of the QTAIM analysis for all 15 complexes is presented in Table S7 of the ESI.† Note that the term “weak H-bond” is used here in the classification of the interactions using Nakanishi's criteria when the evaluated interaction fulfills several but not all of the parameters of a typical H-bond interaction (usually, the ∇^2^*ρ* parameter is lower than the typical range of an H-bond).

**Table 3:** Classification using the Nakanishi criteria of the representative interactions for the complex β-HCH@C4-β-CD.2

Representative interaction	Atoms (β-HCH⋯CD)	Distance (Å)	*ρ* (ea_0_^−3^)	∇^2^*ρ* (ea_0_^−5^)	*H* (au)	*V*/*G*	*ε*	Type of interaction
1	H154⋯O13	3.04	0.0030	0.011	0.0006	−0.73	1.199	vdW
2	H156⋯H9	2.33	0.0052	0.017	0.0007	−0.78	0.418	vdW
3	Cl165⋯H49	2.98	0.0062	0.019	0.0009	−0.77	0.152	vdW
4	Cl165⋯O36	3.04	0.0111	0.040	0.0012	−0.86	0.176	vdW^a^
5	Cl165⋯O15	3.11	0.0104	0.036	0.0010	−0.87	0.229	vdW^a^
6	Cl165⋯H20	2.59	0.0106	0.036	0.0015	−0.80	0.031	Weak H-bond
7	H158⋯O13	2.41	0.0106	0.036	0.0011	−0.87	0.075	Weak H-bond
8	Cl162⋯H112	2.60	0.0101	0.036	0.0016	−0.78	0.077	Weak H-bond
9	Cl165⋯H51	2.69	0.0105	0.034	0.0014	−0.80	0.067	Weak H-bond
10	H157⋯H72	1.84	0.0141	0.048	0.0018	−0.82	0.077	H-bond^b^

a A halogen bond is an interaction with the electron density and energy density of an H-bond between, in these cases, chlorine and oxygen atoms due to the anisotropy of the electronic density of the halogen.

b A dihydrogen bond is an H-bond that is established between two hydrogen atoms with a difference in their partial charges.

**Fig. 10:**
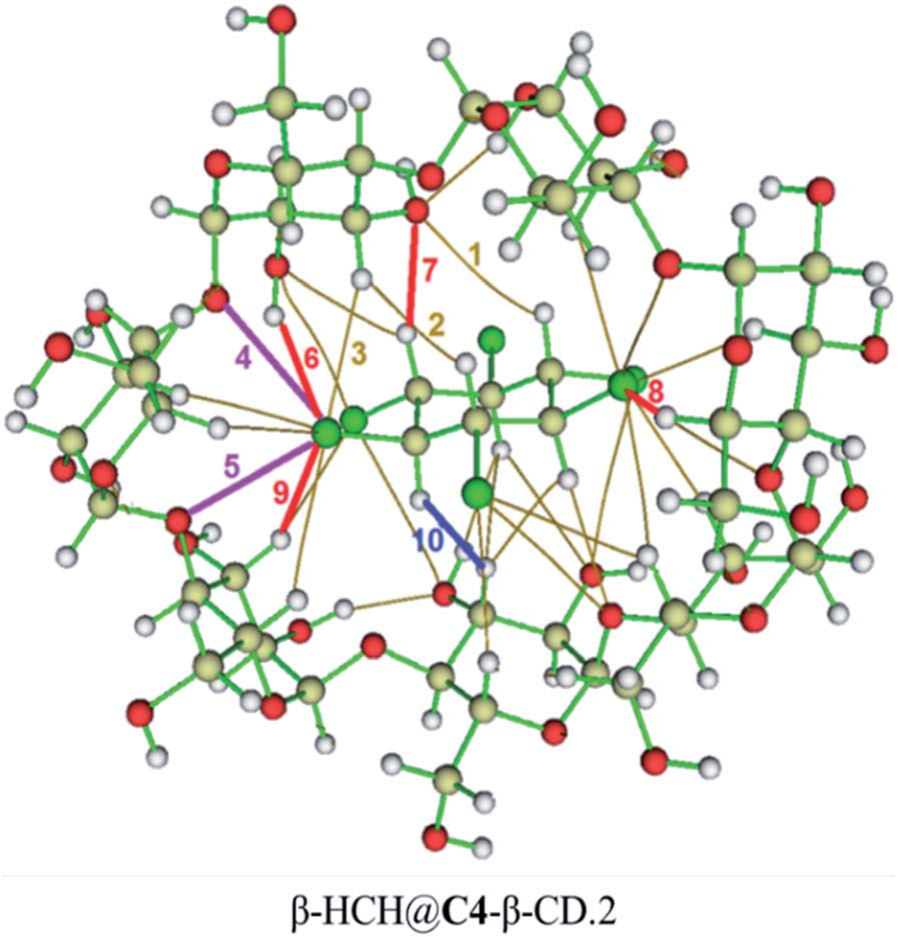
Types of interactions present on the inclusion complexes formed: van der Waals interactions (1, 2 and 3 as examples, in mustard), halogen bonds (4 and 5, in purple), hydrogen bonds (6, 7, 8, and 9, in red), and dihydrogen bonds (10, in blue). Hydrogen atoms are white, carbon atoms are ochre, oxygen atoms are red and chlorine atoms are green. Covalent bonds are green and non-covalent interactions are mustard.

In the complexes, stronger interactions can also be found (Fig. 10), such as H-bonds of different types,^93^ weak H-bonds between the chlorine atoms of the pollutant and the hydrogen atoms of the glycosidic residues, H-bonds between the axial hydrogen atoms of β-HCH and the oxygen atoms of the glucopyranose units, and dihydrogen bonds between the pollutant hydrogens and those of the CD that present significant differences in their partial charges and interatomic distances lower than 2.0 Å. Also, in several cases, it is possible to find halogen bonds due to the anisotropy of the electron density of the chlorine atoms (σ-hole), which is responsible for the contribution of the electrostatic attraction between the chlorine atoms and the oxygens of the cavity.^93^

The presence of a great number of vdW interactions (*i.e.* 78.1% of the interactions for the complex shown in Fig. 10) and several H-bonds with different natures are the driving forces in the stabilization of the complexes. These are in accordance with the MMH results, despite the changes in the molecular geometries and association energies after the DFT calculations.

These results are consistent with QTAIM analyses performed for other guest–host complexes,^34–36^ although in our work, a different criterion for the classification of the interactions was used.

The formation of H-bonds mentioned before increases the stability of several systems. In the complexes where β-CD is the host molecule, a greater number of these interactions are formed. In the case of γ-CD, the size of the cavity allows accommodation of the pollutant inside of it, stabilizing the complexes without the predominant presence of these interactions. Note that in the γ-CD complexes, there are large numbers of dispersive interactions. These are determinant factors for the stabilization of the inclusion complexes.

**Fig. 11:**
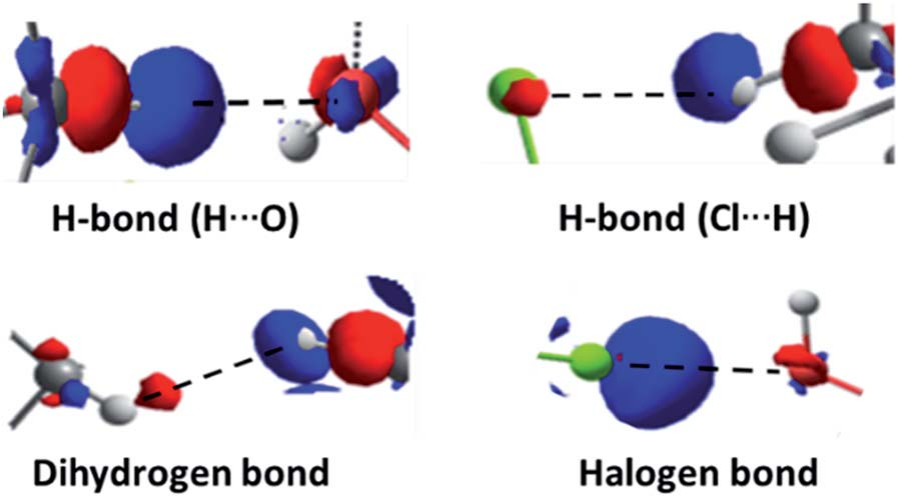
Density-based representations of the different types of electrostatic interaction. Blue represents a decrease of the electron density in the region and red represents an increment. The black dashed lines connect the interacting atoms (isosurface contour value 0.8 × 10^−3^ a.u.).

The results obtained through QTAIM analysis are consistent with the results of the study of the charge transfer process, where interactions such as the previously described H-bonds were detected. Therefore, when comparing the results of the QTAIM analysis and the charge transfer studies for these complexes, representations based on the changes in the electron density in the formation processes of several types of interactions can be made. These representations are shown in Fig. 11.

### Experimental confirmation of the formation of β-HCH@CDs complexes

#### Precipitation of β-HCH@CDs complexes

**Fig. 12:**
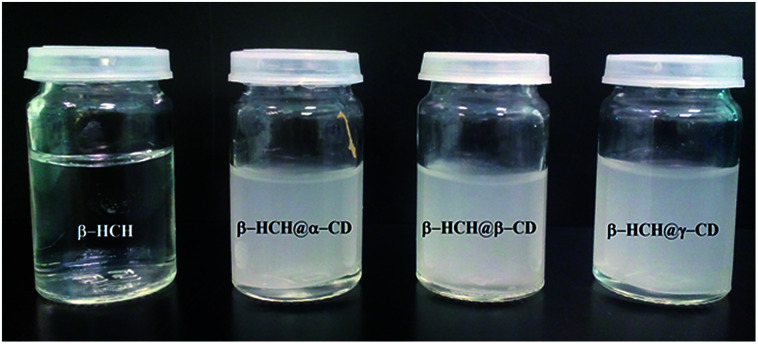
Formation of the non-soluble molecular inclusion complexes β-HCH@CDs.

Fig. 12 shows the results obtained after mixing stoichiometric amounts of host molecules (α-, β- and γ-CDs) and the guest pesticide β-HCH, confirming the precipitation of β-HCH@CDs complexes in all cases. However, after quantification of β-HCH in the liquid phase, it was determined that 90.4%, 98.6% and 99.1% of the pesticide precipitated with α-, β- and γ-CDs, respectively. This result is in agreement with the theoretical findings, which show higher percentages of partial and total occlusion for β-HCH in β- and γ-CDs and higher stability of β-HCH@β-CD and γ-HCH@α-CD complexes than of β-HCH@α-CD complexes.

#### Spectroscopic characterization of inclusion complexes

Resonance Raman (RR) spectra of the CDs (Fig. S3 of the ESI†) show no significant difference between α-, β- and γ-CDs. The CDs show a peak at 2915 cm^−1^ with a shoulder at 2941 cm^−1^, corresponding to aliphatic C–H stretching with asymmetric and symmetric C–H stretching vibrations. This shoulder is more prominent for β and γ-CDs than for α-CD.

**Fig. 13:**
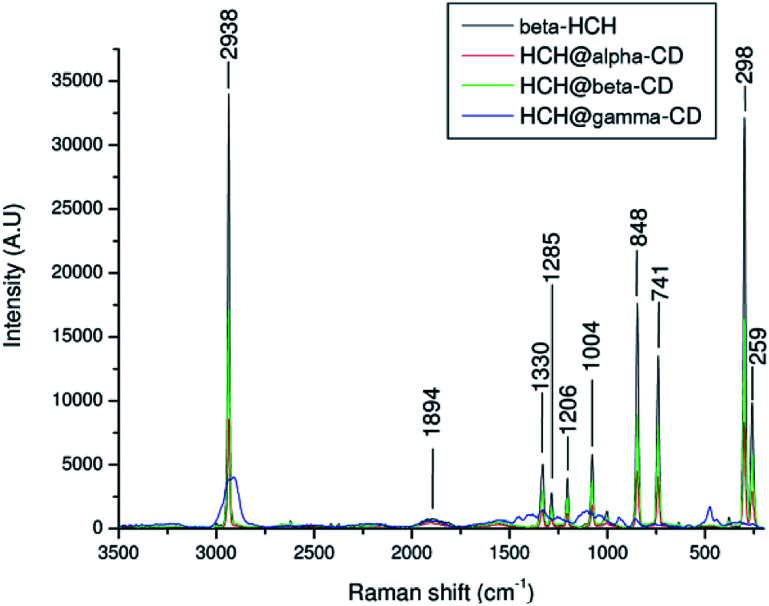
Resonance Raman spectra of β-HCH and of β-HCH@α-CD, β-HCH@β-CD, and β-HCH@γ-CD (α in red, β in green, and γ in blue).

The Raman spectrum of β-HCH shows a prominent peak at 2938 cm^−1^ due to the (C–H) stretching (Fig. 13). The vibrations between 1000 and 1300 cm^−1^, corresponding to the alicyclic chain vibration band (C–C), are exhibited at 1330, 1285, 1206 and 1004 cm^−1^. Peaks at 741 cm^−1^ corresponding to (C–Cl) stretching and two peaks at 298 and 259 cm^−1^ corresponding to (C–C) deformation vibrations are observed. The Raman spectrum of β-HCH@α-CD exhibits the same bands found in the β-HCH spectrum. The RR spectra of β-HCH@β-CD exhibited the same bands as β-HCH with the same intensity (Fig. 13). A peak around 848 cm^−1^ was observed for all the CDs as well as for the different β-HCH@CD complexes. The guest–host complex β-HCH@γ-CD spectrum also shows nearly the same spectrum as β-HCH with some differences, namely the presence of new bands at 1457, 1409, 939 and 475 cm^−1^.

These new absorption bands may be slight guest–host interaction bands (shifts observed when compared with the spectra of the free CDs from 1460 cm^−1^ to 1457 cm^−1^ (Δ*ν* = −3 cm^−1^), from 1393 cm^−1^ to 1409 cm^−1^ (Δ*ν* = +16 cm^−1^), from 939 cm^−1^ to 942 cm^−1^ (Δ*ν* = −3 cm^−1^), and from 477 cm^−1^ to 475 cm^−1^ (Δ*ν* = −2 cm^−1^)). The two peaks at 1457 and 1409 cm^−1^ are provided by the CH_2_ and CH deformations. Meanwhile, the peak at 939 cm^−1^ can be attributed to (C–O–C) stretching^94^ and can also be attributed to coupled vibrational modes of the glycosidic links delocalized around the cyclodextrin ring.^95^ This band was only present in the resonance Raman spectrum of β-HCH@γ-CD, indicating that the bonding of β-HCH with the CDs may be different for γ-CD. This is in agreement with the different degrees of occlusion previously theoretically estimated for the complex formed with each CD (partial occlusion for the complexes formed with α and β-CD and total occlusion for the complex formed with γ-CD). This may account for the difference in the interactions of β-HCH with the inner cavities of the CDs. The assignments of the absorption bands in the Raman spectra are shown in the Table S8 of the ESI.†

The FTIR spectra of the CDs (Fig. S4 of the ESI†) show no significant difference between the α, β, and γ-CDs. The CD spectra show the (O–H) stretching mode around ∼3300 cm^−1^, and two peaks at 2926 and 2917 cm^−1^ correspond to aliphatic C–H stretching^94^ with asymmetric and symmetric CH stretching vibrations.^96^ Bands located from 800 to 1400 cm^−1^ were assigned to (C–O), (C–C), and (C–O–C) stretching at 1153 cm^−1^ and to (O–H) planar angular deformation vibrations.^97^

The ATR-FTIR spectrum of β-HCH (Fig. S5 of the ESI†) shows the presence of symmetric (around 2988 cm^−1^) and asymmetric (around 2975 cm^−1^) C–H sp^3^ stretching vibrations. In the region between 1400 and 1200 cm^−1^, the out-of-plane deformation vibration due to rocking and bending of the 6 (C–H) bonds indicates asymmetric bending around 1341 cm^−1^, deformation vibration of the ring C–H wagging around 1282 cm^−1^, ring deformation vibration between 1101 and 953 cm^−1^ and C–H rocking around 850 cm^−1^. The C–Cl vibrational modes show strong multiple bands around 1200 to 1000 cm^−1^; the band at 778 cm^−1^ presents an intense absorption due to the equatorial C–Cl stretching.

**Fig. 14:**
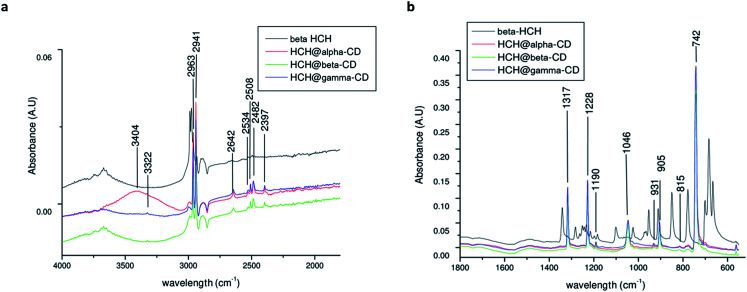
ATR-FTIR spectrum of β-HCH and β-HCH@CDs, (a) at the left in the region of 4000–1800 cm^−1^ and (b) at the right in the region of 1800 to 500 cm^−1^ (β-HCH in black, β-HCH@α-CD in red, β-HCH@β-CD in green, and β-HCH@γ-CD in blue).

The spectra of β-HCH@α-CD (Fig. 14) exhibited more significant new bands at 2963, 2941, 2508, 2642, 2508, 2482, 2397, 1317, 1228, 1190, 1046, 905, and 742 cm^−1^. Furthermore, from the appearance of some peaks (for example, 1046 cm^−1^, corresponding to the *ν*(C–O–C) shifts of the C–O stretching^98^), similar to those of pure α-CD with down-shifting, it is possible to conclude that a complex between α-CD and β-HCH molecules, involving weak forces such as van der Waals interactions and hydrogen bonding,^94^ is formed, which confirms the results of the QTAIM analysis. The spectrum of β-HCH@γ-CD exhibited almost the same bands as those of β-HCH@α-CD and β-HCH@β-CD, with new bands at 2643, 2397 and 931 cm^−1^. The assignments of the absorption bands in ATR-FTIR are shown in Table S9 of the ESI.†

#### Morphological characterization of an inclusion complex

**Fig. 15:**
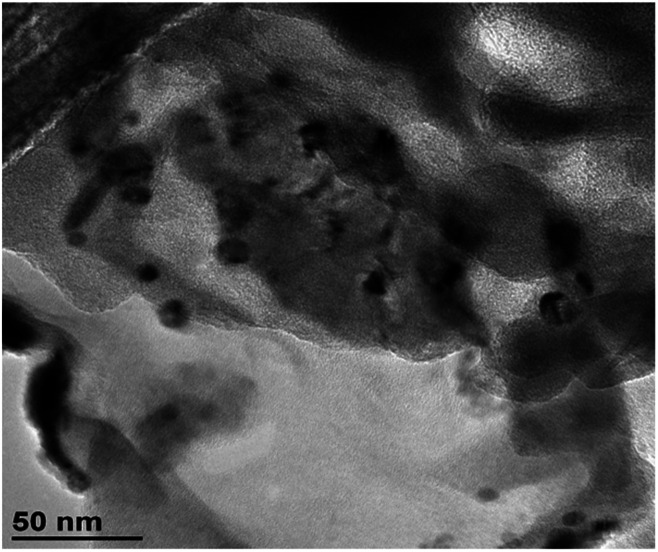
TEM micrograph of the inclusion complex β-HCH@γ-CD.

In order to investigate the morphology of an inclusion complex, β-HCH@γ-CD was investigated by TEM. The TEM micrograph (Fig. 15) shows the presence of agglomerates with an amorphous nature formed of smaller nanoparticles with sizes in the range of 5 to 50 nm.

The results of the present study suggest that it is possible to use CDs for the treatment of water polluted with β-HCH (a suggestion worth checking experimentally on a larger scale). This will enable the development of an alternative process for water treatment, either directly by using CDs for the removal of the pollutant or indirectly through the modification of activated carbon filters or other adsorbent materials. Furthermore, these results could serve as the basis for subsequent studies focusing on the selective separation between β-HCH and other organochlorine pollutants, such as chlordecone.

## Conclusions

The study of the stability and interactions of the inclusion complex β-HCH@CDs suggests the possibility of using CDs in a wide range of alternative methods for the treatment of water polluted with β-HCH.

The configurational space was studied through the multiple minima hypersurface methodology using the semiempirical Hamiltonian PM6-D3H4X. Afterward, some distinctive structures were re-optimized through DFT using the hybrid functional M06-2X with SMD as an implicit solvent model. These studies showed that the most stable complexes are formed, in general terms, when γ-CD is the host molecule. Greater stabilization for conformers that present their primary hydroxyls oriented towards the solution and with greater degrees of occlusion (conformers C1 and C4) were observed. In all experiments, correcting the BSSE was shown to be significant, and this was mitigated in accord with the procedure of Gamboa *et al.*^58^

A conceptual DFT study of several properties, such as charge distribution and the dual descriptor, indicated that it is possible to increase the stability of the complexes by introducing electron-withdrawing substituents into the glucopyranose units of the CDs. The interactions of the pesticide with the CDs in the complexes of interest were studied through the QTAIM methodology, which showed the existence of a large number of dispersive interactions between the pollutant and the inner cavities of the CDs. It also revealed the presence of stronger interactions with the formation of H-bonds of a different nature. At the same time, the QTAIM analysis confirmed the results of the study of the charge transfer process and allowed us to construct density-based representations of the non-van der Waals interactions that occur in the complexes, showing the behavior of the electron density around the involved atoms when those types of interactions were established.

The formation of the complexes was confirmed experimentally, followed by characterization *via* Raman and FTIR spectroscopy that corroborated the theoretical results of this work.

The used methodology could be extensible to other contaminants or systems, such as the study of selective separation cases previously mentioned.

## Conflicts of interest

There are no conflicts to declare.

## Supplementary Material

RA-009-C9RA04431K-s001
