# Supplementary material for: Evaluation of the molecular inclusion process of β-hexachlorocyclohexane in cyclodextrins
Source: RSC Adv. 2019 Sep 2;9(47):27484–99. doi: 10.1039/c9ra04431k (PMC9070783; doi:10.1039/c9ra04431k)
Supplement: RA-009-C9RA04431K-s001 [file RA-009-C9RA04431K-s001.pdf]

Electronic Supplementary Material (ESI) for RSC Advances.
This journal is © The Royal Society of Chemistry 2019

Evaluation of the Molecular Inclusion Process of β-Hexachlorocyclohexane at Cyclodextrins.

*Anthuan Ferino-Pérez[1], Juan José Gamboa-Carballo[1,2], Ronald Ranguin[3], Joëlle Levalois-Grützmacher[2,4], Sarra Gaspard[3], Ramón Alain Miranda-Quintana[5], Melvin Arias[6], Ulises J. Jáuregui-Haza[1,6]**

[1]Instituto Superior de Tecnologías y Ciencias Aplicadas (InSTEC), Universidad de La Habana, La Habana, CP 10600, Cuba

[2]Departament of Chemistry and Applied Biosciences, Laboratory of Inorganic Chemistry, ETH Zürich, CH-8093, Switzerland

[3]Laboratoire COVACHIM M2E, Université des Antilles, Pointe à Pitre, 97157, Guadeloupe, France

[4]Department of Chemistry, Université des Antilles, Pointe à Pitre, 97157, Guadeloupe, France

[5]Department of Chemistry, York University, Toronto, Ontario, Canada

[6]Instituto Tecnológico de Santo Domingo, Área de Ciencias Básicas y Ambientales, Ave. de los Próceres, Santo Domingo, República Dominicana

* **Corresponding author:** ulises.jauregui@intec.edu.do, ulises@instec.cu

Supporting Information

Table S1. Characteristic dimensions of the studied cyclodextrins [1] and of the β-HCH.

<table>
<tr><th colspan="3">cyclodextrins</th><th>α-CD</th><th>β-CD</th><th>γ-CD</th></tr>
<tr><td colspan="3">internal cavity diameter (nm)</td><td>0.47-0.53</td><td>0.60-0.65</td><td>0.75-0.83</td></tr>
<tr><td colspan="3">external cavity diameter (nm)</td><td>1.46</td><td>1.54</td><td>1.75</td></tr>
<tr><td colspan="3">cavity’s height (nm)</td><td>0.79</td><td>0.79</td><td>0.79</td></tr>
<tr><td colspan="3">cavity’s volume (nm3)</td><td>0.174</td><td>0.262</td><td>0.427</td></tr>
<tr><td rowspan="2">β-HCH</td><td>large (nm)</td><td>width (nm)</td><td>superficial area (nm2)</td><td>height (nm)</td><td>volume (nm3)</td></tr>
<tr><td>0.63</td><td>0.53</td><td>0.0278</td><td>0.22</td><td>0.0625</td></tr>
</table>

Table S2. Multiple Minimums Hypersurfaces (MMH) metodology results.

conformers	CDs	energy (eV)	E_{CD+HCH} (eV)	ΔE_{ASSOC} kJ/mol)	ΔS_{ASSOC} (kJ/mol)	ΔA_{ASSOC} (kJ/mol)
A1	α-CD	-13974.7	-16319.3	-48.24	15.67	-52.91
	β-CD	-16303.7	-18648.3	-49.40	17.49	-54.61
	γ-CD	-18632.6	-20977.2	-75.20	4.98	-76.69
A2	α-CD	-13975.0	-16319.6	-50.42	14.36	-54.70
	β-CD	-16304.0	-18648.7	-49.40	20.50	-55.51
	γ-CD	-18632.9	-20977.6	-61.24	12.22	-64.89
A3	α-CD	-13974.6	-16319.2	-50.12	17.92	-55.47
	β-CD	-16303.5	-18648.2	-58.54	8.21	-60.99
	γ-CD	-18632.4	-20977.0	-66.09	11.61	-69.55
A4	α-CD	-13975.1	-16319.8	-48.93	11.33	-52.31
	β-CD	-16304.2	-18648.9	-47.12	19.10	-52.82
	γ-CD	-18633.2	-20977.8	-61.30	8.88	-63.95
B2	α-CD	-13974.1	-16318.7	-75.27	5.13	-76.80
	β-CD	-16302.9	-18647.5	-82.51	7.62	-84.79
	γ-CD	-18631.6	-20976.3	-118.64	0.02	-118.65
B3	α-CD	-13973.7	-16318.3	-77.91	3.31	-78.89
	β-CD	-16302.4	-18647.1	-87.93	11.35	-91.32
	γ-CD	-18631.1	-20975.7	-106.05	17.21	-111.18
C1	α-CD	-13973.9	-16318.5	-49.78	13.31	-53.75
	β-CD	-16302.8	-18647.4	-68.26	17.57	-73.50
	γ-CD	-18631.5	-20976.1	-87.48	9.48	-90.30
C4	α-CD	-13974.4	-16319.0	-48.93	11.33	-52.31
	β-CD	-16303.3	-18647.9	-75.35	9.01	-78.04
	γ-CD	-18632.1	-20976.8	-92.24	3.20	-93.19

Table S3. Energies of the conformers B2, B3, C1 and C4 of the cyclodextrins obtained via DFT M06-2X/SMD and of the β-HCH.

conformers	CDs	E M06-2X/SMD water (kJ/mol)
B2	α-CD	-9618303
	β-CD	-11221380
	γ-CD	-12824441
B3	α-CD	-9618292
	β-CD	-11221369
	γ-CD	-12824429
C1	α-CD	-9618292
	β-CD	-11221353
	γ-CD	-12824400
C4	α-CD	-9618305
	β-CD	-11221368
	γ-CD	-12824416
β-HCH		-7858595

Table S4. Energies at vacuum of studied complexes and reactants (conformers B2, B3, C1 and C4 of the cyclodextrins and the β-HCH) obtained via DFT M06-2X. The latters were calculated using the basis set of the supermolecule.

complexes	$E_{complex}$ (kJ/mol)	E_{CD} (kJ/mol)	$E_{\beta\text{-HCH}}$ (kJ/mol)
**β-HCH@B2-α-CD**	-17476627.0	-7858557.6	-9617973.3
**β-HCH@B2-β-CD**	-19079668.4	-7858557.2	-11220980.2
**β-HCH@B2-γ-CD**	-20682646.8	-7858559.3	-12823960.3
**β-HCH@B3-α-CD**	-17476637.1	-7858556.0	-9617969.1
**β-HCH@B3-β-CD**	-19079681.5	-7858555.8	-11221002.0
**β-HCH@B3-γ-CD**	-20682588.8	-7858558.9	-12824001.4
**β-HCH@C1-α-CD**	-17476659.1	-7858556.3	-9618012.6
**β-HCH@C1-β-CD.1**	-19079678.6	-7858556.9	-11221003.4
**β-HCH@C1-β-CD.2**	-19079686.4	-7858558.6	-11220996.0
**β-HCH@C1-γ-CD.1**	-20682682.2	-7858559.3	-12823997.0
**β-HCH@C1-γ-CD.2**	-20682662.3	-7858559.8	-12823990.3
**β-HCH@C4-α-CD**	-17476621.9	-7858557.4	-9617984.3
**β-HCH@C4-β-CD.1**	-19079624.5	-7858554.7	-11220989.1
**β-HCH@C4-β-CD.2**	-19079635.8	-7858559.9	-11220968.9
**β-HCH@C4-γ-CD**	-20682642.4	-7858560.2	-12823983.0

Table S5. Energies at vacuum of the reactants with the geometry presented on the final complexes.

complexes	E_{CD} (kJ/mol)	$E_{\beta\text{-HCH}}$ (kJ/mol)
**β-HCH@B2-α-CD**	-9617956.0	-7858550.3
**β-HCH@B2-β-CD**	-11220957.3	-7858548.2
**β-HCH@B2-γ-CD**	-12823932.2	-7858549.5
**β-HCH@B3-α-CD**	-9617942.8	-7858547.9
**β-HCH@B3-β-CD**	-11220977.6	-7858546.9
**β-HCH@B3-γ-CD**	-12823978.8	-7858549.9
**β-HCH@C1-α-CD**	-9617980.4	-7858548.4
**β-HCH@C1-β-CD.1**	-11220971.6	-7858548.2
**β-HCH@C1-β-CD.2**	-11220963.3	-7858549.2
**β-HCH@C1-γ-CD.1**	-12823964.0	-7858550.3
**β-HCH@C1-γ-CD.2**	-12823971.5	-7858550.1
**β-HCH@C4-α-CD**	-9617970.2	-7858550.7
**β-HCH@C4-β-CD.1**	-11220976.3	-7858549.0
**β-HCH@C4-β-CD.2**	-11220939.1	-7858550.1
**β-HCH@C4-γ-CD**	-12823956.9	-7858550.0

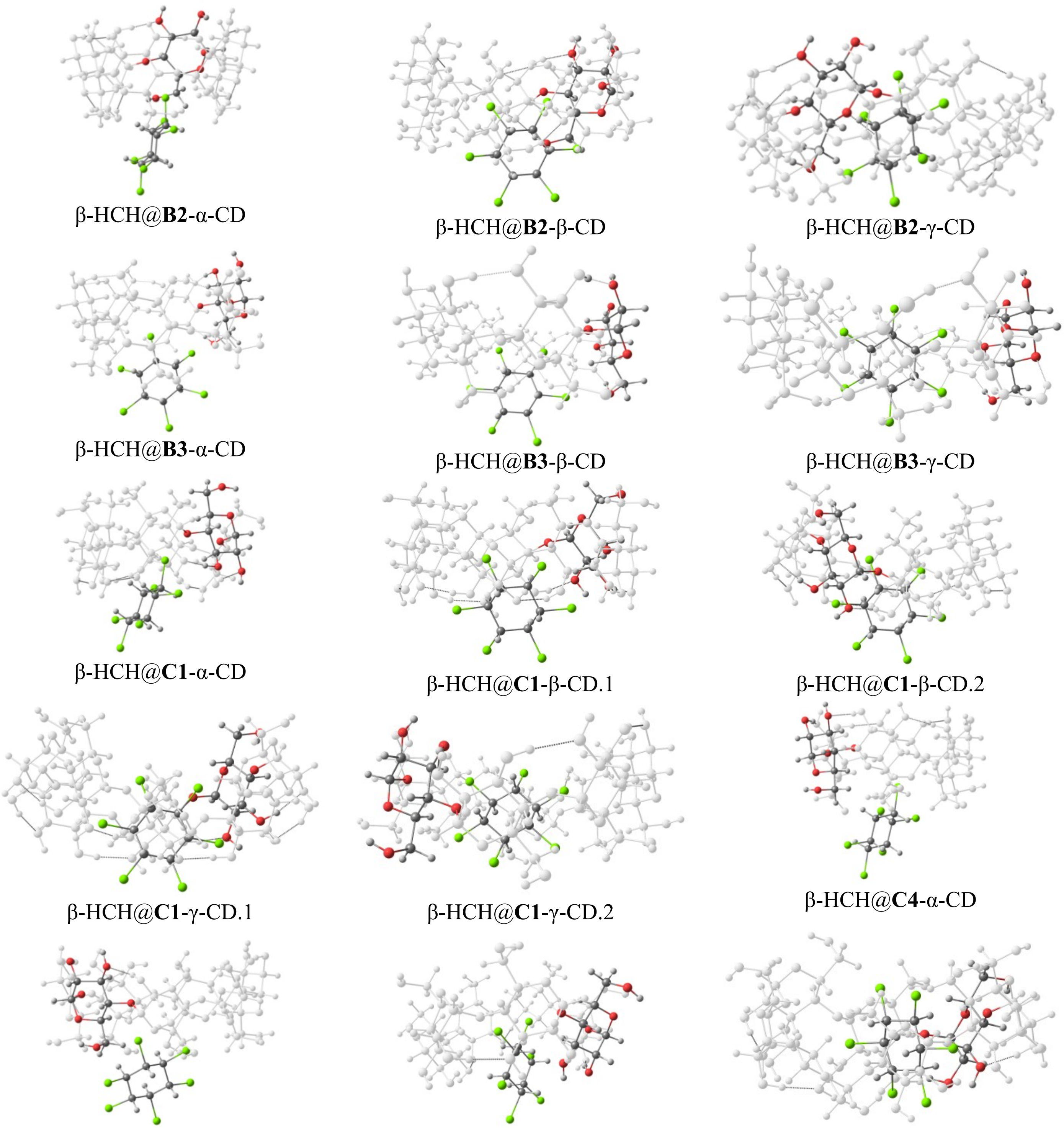

Figure S1. Structures reoptimized by DFT M06-2X of the interest complexes for the conformers B2, B3, C1 and C4. Only a glucopyranose unit is highlighted for simplicity.

Table S6. Condensed dual descriptor for studied molecules.

compounds	#	atoms[a]	q_N[b]	q_{N-1}[b]	q_{N+1}[b]	Δf_A
**β-HCH**	1	C	0.026	0.035	-0.003	0.020
	2	C	0.026	0.035	-0.003	0.020
	3	C	0.026	0.035	-0.003	0.020
	4	C	0.026	0.035	-0.003	0.020
	5	C	0.026	0.035	-0.003	0.020
	6	C	0.026	0.035	-0.003	0.020
	7	H	0.063	0.079	0.030	0.018
	8	H	0.063	0.079	0.030	0.018
	9	H	0.063	0.079	0.030	0.018
	10	H	0.063	0.079	0.030	0.018
	11	H	0.063	0.079	0.030	0.018
	12	H	0.063	0.079	0.030	0.018
	13	Cl	-0.089	0.052	-0.193	-0.037
	14	Cl	-0.089	0.052	-0.193	-0.037
	15	Cl	-0.089	0.053	-0.194	-0.039
	16	Cl	-0.089	0.052	-0.193	-0.037
	17	Cl	-0.089	0.054	-0.194	-0.039
	18	Cl	-0.089	0.052	-0.193	-0.038
**β-HCH@B2-α-CD**	127	C	0.025	0.026	-0.001	0.027
	128	C	0.026	0.026	0.000	0.026
	129	C	0.021	0.021	-0.007	0.028
	130	C	0.028	0.028	-0.001	0.029
	131	C	0.023	0.024	-0.008	0.029
	132	C	0.026	0.026	-0.001	0.026
	133	H	0.061	0.061	0.029	0.032
	134	H	0.063	0.063	0.032	0.031
	135	H	0.035	0.035	0.014	0.020
	136	H	0.058	0.059	0.025	0.032

	137	H	0.040	0.041	0.015	0.024
	138	H	0.062	0.063	0.031	0.031
	139	Cl	-0.091	-0.090	-0.188	0.096
	140	Cl	-0.085	-0.084	-0.179	0.092
	141	Cl	-0.074	-0.073	-0.164	0.089
	142	Cl	-0.014	-0.014	-0.091	0.075
	143	Cl	-0.014	-0.015	-0.095	0.081
	144	Cl	-0.032	-0.031	-0.122	0.088
**β-HCH@B2-β-CD**	148	C	0.025	0.026	-0.005	0.030
	149	C	0.025	0.025	-0.005	0.029
	150	C	0.029	0.030	0.001	0.028
	151	C	0.024	0.024	0.001	0.023
	152	C	0.024	0.024	0.000	0.024
	153	C	0.026	0.026	0.001	0.025
	154	H	0.048	0.048	0.022	0.026
	155	H	0.046	0.047	0.026	0.019
	156	H	0.063	0.064	0.034	0.029
	157	H	0.052	0.052	0.032	0.020
	158	H	0.056	0.056	0.026	0.029
	159	H	0.048	0.048	0.029	0.019
	160	Cl	-0.032	-0.032	-0.127	0.095
	161	Cl	-0.005	-0.004	-0.080	0.075
	162	Cl	-0.031	-0.030	-0.128	0.096
	163	Cl	-0.015	-0.014	-0.092	0.077
	164	Cl	-0.088	-0.088	-0.179	0.090
	165	Cl	-0.090	-0.089	-0.179	0.089
**β-HCH@B2-γ-CD**	169	C	0.023	0.023	0.000	0.023
	170	C	0.017	0.018	-0.002	0.019
	171	C	0.025	0.025	-0.002	0.027
	172	C	0.025	0.026	-0.002	0.027
	173	C	0.027	0.027	-0.007	0.034

	174	C	0.023	0.023	0.000	0.023
	175	H	0.043	0.043	0.016	0.028
	176	H	0.040	0.041	0.022	0.018
	177	H	0.063	0.063	0.023	0.041
	178	H	0.052	0.052	0.029	0.023
	179	H	0.061	0.061	0.010	0.052
	180	H	0.045	0.045	0.026	0.019
	181	Cl	-0.080	-0.079	-0.161	0.080
	182	Cl	-0.022	-0.021	-0.090	0.068
	183	Cl	-0.048	-0.048	-0.107	0.059
	184	Cl	-0.023	-0.023	-0.091	0.068
	185	Cl	-0.035	-0.035	-0.126	0.090
	186	Cl	-0.081	-0.081	-0.181	0.100
**β-HCH@C1-α-CD**	127	C	0.026	0.026	-0.003	0.029
	128	C	0.020	0.021	-0.004	0.025
	129	C	0.025	0.025	-0.002	0.027
	130	C	0.025	0.025	0.000	0.024
	131	C	0.027	0.027	-0.005	0.031
	132	C	0.026	0.027	-0.001	0.025
	133	H	0.063	0.063	0.025	0.037
	134	H	0.043	0.043	0.025	0.017
	135	H	0.062	0.062	0.025	0.036
	136	H	0.044	0.045	0.026	0.018
	137	H	0.049	0.049	0.009	0.039
	138	H	0.049	0.049	0.030	0.018
	139	Cl	-0.064	-0.063	-0.159	0.095
	140	Cl	-0.027	-0.026	-0.107	0.079
	141	Cl	-0.095	-0.094	-0.192	0.096
	142	Cl	-0.072	-0.071	-0.165	0.093
	143	Cl	-0.026	-0.025	-0.116	0.090
	144	Cl	-0.030	-0.030	-0.108	0.077

**β-HCH@C1-β-CD.1**	148	C	0.025	0.026	-0.001	0.025
	149	C	0.027	0.027	0.001	0.026
	150	C	0.027	0.027	-0.003	0.029
	151	C	0.023	0.023	-0.004	0.027
	152	C	0.024	0.024	-0.003	0.026
	153	C	0.022	0.022	-0.002	0.024
	154	H	0.059	0.060	0.025	0.033
	155	H	0.048	0.050	0.028	0.019
	156	H	0.057	0.058	0.023	0.032
	157	H	0.042	0.042	0.025	0.017
	158	H	0.047	0.048	0.020	0.027
	159	H	0.048	0.048	0.029	0.019
	160	Cl	-0.073	-0.070	-0.162	0.087
	161	Cl	-0.093	-0.091	-0.189	0.094
	162	Cl	-0.039	-0.036	-0.127	0.084
	163	Cl	-0.051	-0.049	-0.147	0.093
	164	Cl	-0.039	-0.038	-0.126	0.086
	165	Cl	-0.009	-0.008	-0.089	0.079
**β-HCH@C1-β-CD.2**	148	C	0.024	0.024	-0.004	0.028
	149	C	0.023	0.024	-0.003	0.025
	150	C	0.026	0.026	-0.004	0.029
	151	C	0.023	0.023	-0.002	0.024
	152	C	0.022	0.022	-0.003	0.025
	153	C	0.021	0.021	-0.004	0.025
	154	H	0.056	0.056	0.021	0.034
	155	H	0.042	0.044	0.022	0.018
	156	H	0.054	0.054	0.022	0.032
	157	H	0.042	0.042	0.026	0.016
	158	H	0.038	0.038	0.017	0.021
	159	H	0.044	0.045	0.025	0.019
	160	Cl	-0.078	-0.076	-0.178	0.099

	161	Cl	-0.096	-0.094	-0.195	0.098
	162	Cl	-0.017	-0.017	-0.093	0.075
	163	Cl	-0.061	-0.059	-0.160	0.098
	164	Cl	-0.048	-0.047	-0.136	0.088
	165	Cl	0.008	0.009	-0.058	0.066
**β-HCH@C1-γ-CD.1**	169	C	0.021	0.021	-0.002	0.023
	170	C	0.027	0.027	0.002	0.025
	171	C	0.024	0.024	-0.003	0.027
	172	C	0.026	0.026	-0.003	0.028
	173	C	0.024	0.024	-0.003	0.027
	174	C	0.022	0.022	-0.003	0.025
	175	H	0.044	0.044	0.025	0.018
	176	H	0.047	0.047	0.023	0.024
	177	H	0.044	0.044	0.026	0.018
	178	H	0.057	0.057	0.027	0.030
	179	H	0.043	0.043	0.023	0.019
	180	H	0.041	0.042	0.021	0.021
	181	Cl	-0.074	-0.073	-0.166	0.091
	182	Cl	-0.077	-0.076	-0.170	0.093
	183	Cl	-0.032	-0.032	-0.113	0.081
	184	Cl	-0.056	-0.056	-0.148	0.091
	185	Cl	-0.042	-0.041	-0.131	0.088
	186	Cl	-0.069	-0.069	-0.167	0.098
**β-HCH@C1-γ-CD.2**	169	C	0.026	0.027	-0.001	0.027
	170	C	0.027	0.027	0.002	0.025
	171	C	0.029	0.029	0.001	0.028
	172	C	0.020	0.020	-0.006	0.025
	173	C	0.028	0.029	-0.001	0.029
	174	C	0.026	0.027	0.002	0.024
	175	H	0.062	0.063	0.021	0.040
	176	H	0.056	0.057	0.035	0.020

	177	H	0.065	0.065	0.026	0.038
	178	H	0.038	0.039	0.020	0.018
	179	H	0.059	0.060	0.018	0.040
	180	H	0.053	0.054	0.035	0.017
	181	Cl	-0.057	-0.054	-0.140	0.081
	182	Cl	-0.025	-0.024	-0.100	0.074
	183	Cl	-0.034	-0.033	-0.110	0.076
	184	Cl	-0.047	-0.046	-0.134	0.086
	185	Cl	-0.022	-0.021	-0.103	0.080
	186	Cl	-0.048	-0.047	-0.134	0.084

[a] Only the pesticide atoms are showed in the case of the complexes to ease the comparison.

[b] The atom charges were estimated using the Hirshfeld population analysis.

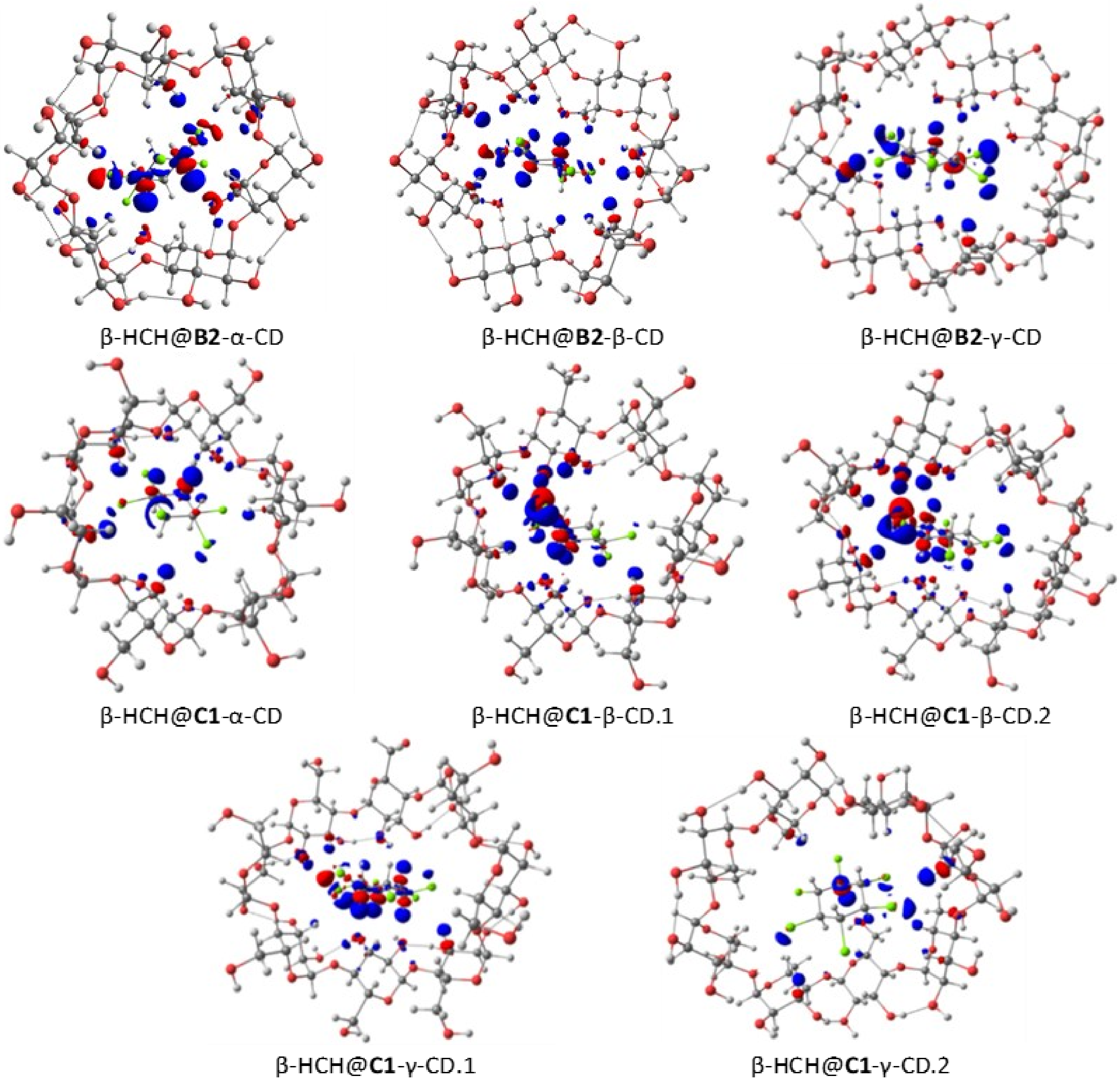

Figure S2. Charge transfer process during the formation of the complexes of the conformers B2 and C1 (isosurface contour value $0.8 \cdot 10^{-3}$ a.u). The complexes are shown from an upper view.

Table S7. QTAIM analysis of the inclusion complexes β-HCH@CD.

complexes	interaction number	atoms (β-HCH...CD)	d (Å)	ρ (ea_0^{-3})	$\nabla^2\rho$ (ea_0^{-5})	H (au)	V/G	ε	interaction type
**β-HCH@B2-α-CD**	1	Cl143…H105	2.64	0.0097	0.035	0.00126	-0.83	0.32	vdW
	2	Cl143…H70	3.04	0.0062	0.020	0.00089	-0.78	0.49	vdW
	3	Cl143…H81	2.80	0.0086	0.029	0.00127	-0.79	0.25	vdW
	4	Cl143…H102	2.93	0.0082	0.028	0.00120	-0.79	0.49	vdW
	5	Cl143...H91	3.04	0.0062	0.019	0.00085	-0.78	0.67	vdW
	6	Cl144…H112	3.34	0.0029	0.009	0.00051	-0.69	0.12	vdW
	7	Cl142…H18	3.09	0.0053	0.017	0.00075	-0.79	0.19	vdW
	8	Cl141…H18	2.94	0.0065	0.020	0.00095	-0.77	0.05	vdW
	9	Cl144...H70	3.23	0.0037	0.011	0.00059	-0.72	0.28	vdW
	10	Cl144...H49	3.01	0.0055	0.017	0.00088	-0.74	0.12	vdW
	11	Cl144…H60	3.24	0.0042	0.013	0.00062	-0.76	0.67	vdW
	12	Cl142…H60	2.85	0.0075	0.024	0.00115	-0.76	0.13	vdW
	13	Cl142…H39	2.77	0.0085	0.032	0.00144	-0.78	0.43	vdW
	14	Cl142…H28	2.94	0.0069	0.022	0.00096	-0.79	0.34	vdW
	15	Cl142…H42	2.66	0.0100	0.036	0.00134	-0.82	0.19	Weak H-bond
	16	Cl142…H7	3.23	0.0042	0.013	0.00061	-0.77	0.23	vdW
	17	Cl144…H7	3.41	0.0027	0.008	0.00043	-0.71	0.22	vdW
	18	Cl144…O80	3.82	0.0033	0.010	0.00053	-0.75	0.19	vdW
	19	H136…O80	3.08	0.0031	0.011	0.00053	-0.76	0.32	vdW
	20	H135…O17	2.06	0.0204	0.078	0.00196	-0.89	0.07	H-bond
	21	H137…O122	2.18	0.0158	0.060	0.00194	-0.85	0.09	H-bond
**β-HCH@B2-β-CD**	1	Cl162...H49	3.14	0.0043	0.013	0.00069	-0.73	0.16	vdW
	2	Cl162…H70	2.72	0.0090	0.030	0.00134	-0.78	0.09	vdW
	3	Cl162…H146	2.95	0.0065	0.020	0.00094	-0.77	0.10	vdW
	4	H156…H146	2.45	0.0048	0.016	0.00082	-0.74	1.05	vdW
	5	Cl163…H81	2.86	0.0074	0.024	0.00114	-0.76	0.12	vdW
	6	Cl163…H49	3.00	0.0064	0.020	0.00084	-0.79	0.15	vdW

	7	Cl163…H63	2.57	0.0115	0.042	0.00145	-0.84	0.25	Weak H-bond
	8	Cl163…H39	2.99	0.0062	0.019	0.00086	-0.77	0.09	vdW
	9	H155…H28	2.22	0.0065	0.021	0.00097	-0.78	0.26	vdW
	10	H155…O38	2.41	0.0107	0.036	0.00108	-0.86	0.11	Weak H-bond
	11	H154…H91	2.76	0.0023	0.008	0.00045	-0.70	0.41	vdW
	12	H154…O120	2.43	0.0104	0.034	0.00099	-0.87	0.06	Weak H-bond
	13	H158…O120	2.92	0.0040	0.014	0.00060	-0.79	0.35	vdW
	14	H157…O38	2.61	0.0067	0.023	0.00082	-0.83	0.12	vdW
	15	Cl160…H7	2.89	0.0072	0.023	0.00109	-0.76	0.12	vdW
	16	Cl160…O139	3.62	0.0039	0.014	0.00066	-0.76	0.26	vdW
	17	Cl160…H133	3.25	0.0029	0.009	0.00053	-0.71	0.38	vdW
	18	Cl160…H18	3.23	0.0046	0.013	0.00056	-0.80	0.58	vdW
	19	Cl160…H131	2.89	0.0069	0.022	0.00105	-0.76	0.01	vdW
	20	Cl160…O118	3.76	0.0034	0.011	0.00057	-0.75	0.67	vdW
	21	Cl160…H110	2.97	0.0058	0.018	0.00095	-0.74	0.07	vdW
	22	H159…O38	2.58	0.0075	0.024	0.00080	-0.85	0.06	vdW
	23	H159…H18	2.38	0.0068	0.024	0.00105	-0.79	2.08	vdW
	24	Cl161…H18	2.78	0.0086	0.028	0.00124	-0.79	0.09	vdW
	25	Cl161…O120	3.36	0.0066	0.024	0.00096	-0.81	1.32	vdW
	26	Cl161…H110	3.02	0.0067	0.021	0.00090	-0.80	0.51	vdW
	27	Cl161…H121	2.85	0.0087	0.029	0.00123	-0.80	0.26	vdW
	28	Cl161…H145	2.64	0.0094	0.034	0.00129	-0.82	0.32	vdW
	29	Cl161…H131	2.88	0.0081	0.025	0.00104	-0.80	0.12	vdW
**β-HCH@B2-γ-CD**	1	Cl185…H93	3.11	0.0044	0.014	0.00075	-0.72	0.12	vdW
	2	Cl185…O78	3.63	0.0042	0.014	0.00066	-0.78	1.92	vdW
	3	Cl185…H72	2.92	0.0053	0.018	0.00093	-0.74	0.04	vdW
	4	Cl185…O57	3.53	0.0048	0.016	0.00070	-0.79	0.12	vdW
	5	Cl185…H49	3.12	0.0045	0.013	0.00072	-0.73	0.01	vdW
	6	Cl185…H70	2.76	0.0089	0.029	0.00129	-0.78	0.06	vdW
	7	Cl182…H112	3.20	0.0044	0.013	0.00065	-0.75	0.31	vdW

	8	Cl182…H123	2.93	0.0060	0.020	0.00096	-0.76	0.20	vdW
	9	Cl182…H91	2.74	0.0085	0.028	0.00134	-0.77	0.09	vdW
	10	Cl182…O101	3.28	0.0070	0.025	0.00094	-0.82	0.06	vdW
	11	Cl182…H70	2.74	0.0096	0.031	0.00134	-0.79	0.15	vdW
	12	Cl182…O80	3.30	0.0074	0.027	0.00096	-0.83	0.32	vdW
	13	H180…O80	2.50	0.0094	0.031	0.00089	-0.87	0.10	vdW
	14	H180…H49	2.34	0.0051	0.016	0.00074	-0.78	0.15	vdW
	15	H180…H60	2.58	0.0035	0.012	0.00064	-0.74	1.26	vdW
	16	Cl186…H30	3.21	0.0035	0.011	0.00066	-0.69	0.15	vdW
	17	H179…H114	3.19	0.0007	0.003	0.00021	-0.52	1.67	vdW
	18	H175…O143	2.37	0.0113	0.040	0.00126	-0.85	0.08	Weak H-bond
	19	Cl181…H60	3.54	0.0021	0.006	0.00037	-0.69	0.88	vdW
	20	Cl181…H144	3.17	0.0043	0.014	0.00068	-0.75	0.11	vdW
	21	H178…H49	2.56	0.0035	0.011	0.00053	-0.76	0.27	vdW
	22	H178…O36	3.02	0.0029	0.011	0.00060	-0.71	0.05	vdW
	23	H178…O59	3.09	0.0032	0.011	0.00054	-0.74	0.73	vdW
	24	H176…O59	2.42	0.0096	0.033	0.00115	-0.84	0.12	vdW
	25	Cl183…H144	3.07	0.0055	0.017	0.00075	-0.78	0.29	vdW
	26	Cl183…H133	2.98	0.0065	0.020	0.00089	-0.78	0.16	vdW
	27	Cl183…O164	2.94	0.0122	0.047	0.00150	-0.85	0.12	vdWa
	28	Cl183…H154	3.04	0.0051	0.016	0.00084	-0.74	0.29	vdW
	29	Cl184…H28	2.78	0.0091	0.029	0.00133	-0.78	0.02	vdW
	30	Cl184…O15	3.15	0.0086	0.031	0.00107	-0.84	0.13	vdW
	31	Cl184…H7	2.70	0.0083	0.029	0.00142	-0.76	0.00	vdW
	32	Cl184…H9	2.90	0.0058	0.019	0.00101	-0.74	0.03	vdW
**β-HCH@B3-α-CD**	1	Cl143…H60	3.11	0.0051	0.015	0.00072	-0.77	0.25	vdW
	2	Cl143…H49	3.08	0.0052	0.016	0.00075	-0.77	0.19	vdW
	3	Cl143…O80	2.91	0.0135	0.050	0.00145	-0.87	0.05	vdWa
	4	Cl143…H70	2.77	0.0080	0.027	0.00124	-0.77	0.02	vdW
	5	Cl143…H102	2.74	0.0096	0.031	0.00136	-0.79	0.03	vdW

	6	Cl143…H91	2.94	0.0067	0.021	0.00096	-0.78	0.31	vdW
	7	Cl140…H100	2.84	0.0075	0.025	0.00120	-0.76	0.08	vdW
	8	Cl140…O122	3.36	0.0061	0.022	0.00086	-0.82	0.06	vdW
	9	H137…O122	2.44	0.0105	0.035	0.00105	-0.86	0.17	Weak H-bond
	10	H133…O122	2.34	0.0119	0.042	0.00136	-0.85	0.09	Weak H-bond
	11	Cl144…H49	3.15	0.0044	0.013	0.00067	-0.75	0.17	vdW
	12	H136…O59	2.38	0.0107	0.038	0.00123	-0.85	0.13	Weak H-bond
	13	Cl144…H28	2.82	0.0077	0.025	0.00121	-0.76	0.08	vdW
	14	Cl144…H7	2.76	0.0088	0.028	0.00129	-0.78	0.06	vdW
	15	Cl144…H112	3.22	0.0038	0.011	0.00061	-0.72	0.09	vdW
	16	Cl141…H18	3.31	0.0036	0.011	0.00053	-0.76	4.13	vdW
	17	H135…O17	2.22	0.0151	0.055	0.00159	-0.87	0.05	Weak H-bond
	18	Cl142…H18	2.83	0.0087	0.028	0.00117	-0.80	0.22	vdW
	19	Cl142…H7	2.87	0.0082	0.027	0.00107	-0.81	0.21	vdW
	20	Cl142…H42	2.47	0.0137	0.050	0.00171	-0.84	0.21	Weak H-bond
	21	Cl142…H28	3.03	0.0064	0.020	0.00087	-0.79	0.66	vdW
	22	Cl142…H39	2.91	0.0078	0.031	0.00144	-0.77	6.79	vdW
**β-HCH@B3-β-CD**	1	Cl162…H60	2.93	0.0068	0.021	0.00095	-0.78	0.09	vdW
	2	Cl162…H70	2.96	0.0069	0.022	0.00091	-0.80	0.20	vdW
	3	Cl162…H84	2.61	0.0108	0.039	0.00140	-0.83	0.22	Weak H-bond
	4	Cl162…H81	2.95	0.0069	0.027	0.00128	-0.77	1.52	vdW
	5	Cl162…H146	2.79	0.0084	0.027	0.00126	-0.77	0.11	vdW
	6	Cl163…H70	3.06	0.0050	0.015	0.00080	-0.74	0.11	vdW
	7	Cl163…H146	3.18	0.0048	0.015	0.00066	-0.78	1.94	vdW
	8	Cl163…H91	2.68	0.0098	0.033	0.00142	-0.79	0.10	vdW
	9	Cl163…H121	2.96	0.0064	0.019	0.00094	-0.76	0.12	vdW
	10	H155…H121	2.39	0.0052	0.017	0.00082	-0.76	0.54	vdW
	11	H157…H119	2.80	0.0025	0.009	0.00052	-0.70	0.58	vdW
	12	H157…H110	2.70	0.0026	0.009	0.00048	-0.71	0.04	vdW
	13	H157…O141	2.59	0.0079	0.025	0.00074	-0.86	0.06	vdW

	14	H154…H60	2.42	0.0047	0.017	0.00080	-0.78	3.70	vdW
	15	H154…O59	2.63	0.0066	0.023	0.00081	-0.83	0.12	vdW
	16	H156…H49	2.21	0.0069	0.022	0.00098	-0.79	0.31	vdW
	17	H156…O59	2.36	0.0118	0.040	0.00120	-0.87	0.10	Weak H-bond
	18	H158…O59	2.56	0.0078	0.025	0.00081	-0.85	0.07	vdW
	19	H158…H39	2.35	0.0072	0.026	0.00106	-0.80	2.14	vdW
	20	Cl165…H28	2.89	0.0072	0.023	0.00109	-0.76	0.11	vdW
	21	Cl165…H39	3.27	0.0043	0.013	0.00054	-0.79	1.45	vdW
	22	Cl165…O15	3.63	0.0038	0.013	0.00064	-0.76	0.18	vdW
	23	Cl165…H7	2.98	0.0059	0.018	0.00090	-0.76	0.15	vdW
	24	Cl165…H9	3.37	0.0023	0.008	0.00044	-0.69	0.60	vdW
	25	Cl165…H131	3.07	0.0047	0.015	0.00080	-0.72	0.13	vdW
	26	Cl164…H39	2.68	0.0103	0.034	0.00141	-0.80	0.06	Weak H-bond
	27	Cl164…H7	2.78	0.0091	0.030	0.00124	-0.80	0.05	vdW
	28	Cl164…O17	2.82	0.0163	0.062	0.00168	-1.14	0.05	vdW[a]
	29	Cl164…O141	3.34	0.0068	0.024	0.00093	-0.82	0.38	vdW
	30	Cl164…H131	3.08	0.0057	0.018	0.00078	-0.79	0.61	vdW
	31	Cl164…H142	2.87	0.0081	0.027	0.00117	-0.79	0.23	vdW
	32	H159…O141	3.08	0.0030	0.011	0.00056	-0.75	1.29	vdW
**β-HCH@B3-γ-CD**	1	H175…H154	2.86	0.0016	0.006	0.00038	-0.64	0.07	vdW
	2	H175…O17	2.85	0.0044	0.015	0.00063	-0.80	0.11	vdW
	3	Cl183…H7	3.21	0.0035	0.011	0.00064	-0.69	0.04	vdW
	4	Cl183…H28	2.62	0.0104	0.036	0.00157	-0.79	0.08	Weak H-bond
	5	Cl183…H49	3.25	0.0037	0.011	0.00057	-0.74	0.08	vdW
	6	Cl181…H39	3.44	0.0027	0.008	0.00046	-0.71	0.08	vdW
	7	Cl181…H60	2.95	0.0065	0.021	0.00103	-0.76	0.30	vdW
	8	Cl181…H81	3.04	0.0059	0.018	0.00083	-0.77	0.24	vdW
	9	H176…H81	2.29	0.0055	0.018	0.00084	-0.77	0.13	vdW
	10	H178…H70	2.95	0.0014	0.005	0.00033	-0.62	1.03	vdW
	11	H178…O101	3.03	0.0037	0.012	0.00054	-0.78	0.19	vdW

	12	H178…H91	2.42	0.0042	0.013	0.00065	-0.76	0.08	vdW
	13	H180…H79	2.60	0.0029	0.010	0.00055	-0.71	0.45	vdW
	14	H180…O101	2.49	0.0088	0.029	0.00096	-0.85	0.07	vdW
	15	Cl185…O101	3.38	0.0063	0.022	0.00087	-0.81	0.25	vdW
	16	Cl185…H102	2.96	0.0068	0.023	0.00102	-0.78	0.18	vdW
	17	Cl185…H91	3.12	0.0053	0.016	0.00071	-0.78	0.43	vdW
	18	Cl185…O122	3.10	0.0096	0.034	0.00112	-0.85	0.04	vdW
	19	Cl185…H112	3.02	0.0056	0.017	0.00083	-0.76	0.05	vdW
	20	Cl185…H144	2.91	0.0068	0.022	0.00103	-0.76	0.15	vdW
	21	Cl186…H114	2.79	0.0074	0.025	0.00121	-0.76	0.09	vdW
	22	Cl186…H112	2.94	0.0062	0.020	0.00095	-0.76	0.05	vdW
	23	Cl186…O120	3.18	0.0080	0.029	0.00102	-0.84	0.06	vdW
	24	Cl186…H135	3.15	0.0042	0.013	0.00069	-0.73	0.20	vdW
	25	Cl186…H133	2.95	0.0065	0.020	0.00095	-0.76	0.01	vdW
	26	H179…O164	2.42	0.0103	0.035	0.00110	-0.86	0.11	Weak H-bond
	27	Cl182…H165	3.01	0.0056	0.017	0.00086	-0.75	0.07	vdW
**β-HCH@C1-α-CD**	1	Cl140…H30	3.20	0.0043	0.012	0.00059	-0.77	0.31	vdW
	2	Cl140…O35	3.40	0.0061	0.021	0.00088	-0.80	0.23	vdW
	3	Cl140…H51	2.57	0.0104	0.037	0.00164	-0.79	0.05	Weak H-bond
	4	Cl140…H72	2.88	0.0070	0.022	0.00106	-0.76	0.03	vdW
	5	Cl139…O76	3.81	0.0030	0.010	0.00055	-0.72	0.23	vdW
	6	Cl139…H72	3.47	0.0030	0.009	0.00042	-0.77	0.93	vdW
	7	Cl139…O77	3.56	0.0050	0.017	0.00076	-0.78	0.49	vdW
	8	Cl139...O97	3.47	0.0060	0.020	0.00079	-0.81	0.57	vdW
	9	H138…O97	2.63	0.0076	0.028	0.00095	-0.84	1.43	vdW
	10	H138…H93	2.07	0.0094	0.001	0.00129	-0.81	0.22	vdW
	11	Cl142…O118	3.80	0.0035	0.012	0.00060	-0.74	0.86	vdW
	12	Cl142…O98	3.38	0.0063	0.022	0.00088	-0.81	0.51	vdW
	13	H134…O97	2.28	0.0140	0.048	0.00137	-0.87	0.22	Weak H-bond
	14	H134…O98	2.88	0.0051	0.018	0.00073	-0.80	0.18	vdW

	15	H136…H93	2.48	0.0046	0.014	0.00057	-0.80	0.69	vdW
	16	H136…O98	2.50	0.0093	0.030	0.00090	-0.86	0.04	vdW
	17	Cl144…H9	2.87	0.0079	0.026	0.00111	-0.80	0.29	vdW
	18	Cl144…O13	3.16	0.0092	0.034	0.00117	-0.84	0.46	vdW
	19	Cl144…H114	2.65	0.0112	0.037	0.00146	-0.81	0.06	Weak H-bond
	20	Cl144…O119	3.48	0.0059	0.020	0.00082	-0.80	0.31	vdW
	21	Cl143…H30	2.90	0.0069	0.022	0.00100	-0.77	0.12	vdW
	22	Cl143…O15	3.76	0.0034	0.011	0.00057	-0.75	0.87	vdW
	23	Cl143…H9	2.91	0.0066	0.021	0.00102	-0.75	0.09	vdW
	24	Cl143…O120	3.74	0.0035	0.012	0.00057	-0.75	0.46	vdW
	25	Cl143…H114	3.12	0.0048	0.015	0.00070	-0.76	0.25	vdW
	26	Cl143…H112	3.44	0.0023	0.007	0.00042	-0.70	0.29	vdW
	27	Cl143…O99	3.85	0.0030	0.010	0.00049	-0.74	0.30	vdW
	28	Cl143…H93	3.13	0.0046	0.014	0.00074	-0.73	0.65	vdW
**β-HCH@C1-β-CD.1**	1	Cl165…H41	2.66	0.0092	0.031	0.00137	-0.78	0.06	vdW
	2	Cl165…O36	3.29	0.0076	0.025	0.00084	-0.85	0.14	vdW
	3	Cl165…H49	2.83	0.0062	0.022	0.00111	-0.74	0.05	vdW
	4	Cl165…H51	2.92	0.0069	0.021	0.00095	-0.78	0.10	vdW
	5	Cl165…O57	3.41	0.0061	0.021	0.00078	-0.82	0.11	vdW
	6	Cl165…H70	3.20	0.0038	0.012	0.00062	-0.73	0.15	vdW
	7	Cl165…H72	3.16	0.0044	0.013	0.00064	-0.76	0.33	vdW
	8	Cl164…H41	2.88	0.0067	0.024	0.00104	-0.79	0.06	vdW
	9	Cl164…H51	2.98	0.0060	0.019	0.00091	-0.76	0.06	vdW
	10	Cl164…O76	3.43	0.0055	0.021	0.00096	-0.78	2.73	vdW
	11	Cl164…H72	2.87	0.0077	0.024	0.00107	-0.79	0.01	vdW
	12	Cl164…O97	3.58	0.0047	0.016	0.00072	-0.79	0.60	vdW
	13	H158…O35	2.63	0.0077	0.027	0.00084	-0.86	0.55	vdW
	14	H158…O34	2.92	0.0038	0.013	0.00066	-0.75	0.21	vdW
	15	H156…O34	2.93	0.0037	0.013	0.00065	-0.75	0.02	vdW
	16	H156…H9	2.84	0.0016	0.006	0.00039	-0.65	0.20	vdW

	17	H157…O97	2.30	0.0135	0.049	0.00139	-0.87	0.21	Weak H-bond
	18	H157…H93	1.99	0.0113	0.042	0.00162	-0.82	0.94	Weak H-bond[b]
	19	H159…O97	2.36	0.0113	0.040	0.00130	-0.85	0.17	Weak H-bond
	20	H155…H93	2.51	0.0043	0.013	0.00055	-0.80	0.46	vdW
	21	H155…O98	2.66	0.0070	0.022	0.00078	-0.84	0.08	vdW
	22	H155…O116	2.94	0.0047	0.017	0.00071	-0.80	1.11	vdW
	23	Cl160…O116	3.78	0.0035	0.012	0.00060	-0.74	0.46	vdW
	24	Cl160…O98	3.56	0.0046	0.016	0.00073	-0.78	0.54	vdW
	25	Cl163…O99	3.93	0.0026	0.008	0.00045	-0.73	0.54	vdW
	26	Cl163…H112	3.14	0.0046	0.014	0.00067	-0.76	0.45	vdW
	27	Cl163…H110	3.50	0.0021	0.006	0.00038	-0.69	0.97	vdW
	28	Cl163…H131	3.17	0.0033	0.011	0.00065	-0.69	0.11	vdW
	29	Cl162…O13	3.73	0.0032	0.011	0.00061	-0.72	0.19	vdW
	30	Cl162…H133	3.18	0.0036	0.011	0.00064	-0.71	0.20	vdW
	31	Cl162…O117	3.52	0.0054	0.019	0.00081	-0.79	0.26	vdW
	32	Cl162…H112	2.67	0.0109	0.036	0.00143	-0.81	0.07	Weak H-bond
**β-HCH@C1-β-CD.2**	1	Cl165…H41	2.56	0.0112	0.037	0.00144	-0.82	0.05	Weak H-bond
	2	Cl165…O36	3.16	0.0096	0.032	0.00094	-0.87	0.19	vdW
	3	Cl165…H49	2.68	0.0084	0.030	0.00144	-0.77	0.03	vdW
	4	Cl165…H51	2.76	0.0089	0.029	0.00124	-0.79	0.14	vdW
	5	Cl165…O57	3.14	0.0096	0.157	0.00103	-0.86	0.16	vdW
	6	Cl165…H70	3.05	0.0053	0.016	0.00080	-0.76	0.16	vdW
	7	Cl165…H72	2.80	0.0085	0.027	0.00117	-0.79	0.08	vdW
	8	Cl164…H41	2.98	0.0057	0.021	0.00091	-0.79	0.51	vdW
	9	Cl164…H72	2.81	0.0085	0.027	0.00114	-0.80	0.05	vdW
	10	Cl164…O97	3.53	0.0052	0.018	0.00076	-0.79	0.38	vdW
	11	H157…H93	1.91	0.0135	0.047	0.00169	-0.83	0.22	Weak H-bond[b]
	12	H159…O97	2.34	0.0123	0.042	0.00125	-0.87	0.10	Weak H-bond
	13	H158…O35	2.66	0.0075	0.025	0.00079	-0.86	0.49	vdW

	14	H158…O34	2.43	0.0096	0.033	0.00113	-0.84	0.09	vdW
	15	H156…H30	2.34	0.0054	0.018	0.00073	-0.80	0.48	vdW
	16	H156…O34	2.88	0.0045	0.016	0.00068	-0.80	0.80	vdW
	17	H154…O34	3.04	0.0033	0.011	0.00055	-0.75	0.84	vdW
	18	Cl163…O99	3.89	0.0028	0.009	0.00047	-0.73	0.28	vdW
	19	Cl163…H110	3.22	0.0036	0.011	0.00062	-0.71	0.10	vdW
	20	H155…H93	2.24	0.0068	0.021	0.00083	-0.81	0.19	vdW
	21	H155…O98	2.71	0.0063	0.020	0.00071	-0.83	0.05	vdW
	22	H155…O99	2.86	0.0046	0.015	0.00065	-0.79	0.03	vdW
	23	Cl160…O98	3.57	0.0044	0.015	0.00069	-0.78	0.18	vdW
	24	Cl162…H9	2.94	0.0065	0.020	0.00097	-0.76	0.01	vdW
	25	Cl162…O139	3.40	0.0055	0.020	0.00082	-0.80	0.04	vdW
	26	Cl162…H133	2.52	0.0115	0.041	0.00172	-0.80	0.05	Weak H-bond
	27	Cl162…H131	3.05	0.0049	0.016	0.00078	-0.75	0.07	vdW
	28	Cl162…O118	3.32	0.0070	0.024	0.00086	-0.83	0.09	vdW
	29	Cl162…H112	2.88	0.0068	0.022	0.00110	-0.75	0.03	vdW
**β-HCH@C1-γ-CD.1**	1	Cl183…H51	2.87	0.0079	0.025	0.00100	-0.81	0.13	vdW
	2	Cl183…H62	2.68	0.0083	0.029	0.00148	-0.75	0.14	vdW
	3	Cl183…O97	3.46	0.0048	0.018	0.00083	-0.77	0.18	vdW
	4	Cl183…H93	3.04	0.0055	0.017	0.00079	-0.77	0.03	vdW
	5	Cl183…O118	3.35	0.0069	0.023	0.00084	-0.83	0.02	vdW
	6	Cl184…H70	2.85	0.0059	0.020	0.00111	-0.72	0.07	vdW
	7	Cl184…H51	3.09	0.0049	0.015	0.00075	-0.74	0.12	vdW
	8	Cl184…O57	3.82	0.0029	0.010	0.00053	-0.72	1.39	vdW
	9	H176…H51	2.37	0.0059	0.020	0.00077	-0.82	1.87	vdW
	10	H176…O55	2.94	0.0047	0.018	0.00072	-0.80	1.18	vdW
	11	H176…O35	2.62	0.0076	0.024	0.00076	-0.85	0.06	vdW
	12	Cl181…O35	3.31	0.0072	0.026	0.00103	-0.82	0.59	vdW
	13	H177…O118	2.34	0.0126	0.044	0.00124	-0.87	0.11	Weak H-bond
	14	H175…O118	2.34	0.0122	0.043	0.00141	-0.85	0.17	Weak H-bond

	15	H178…H30	2.32	0.0047	0.016	0.00080	-0.74	0.01	vdW
	16	H180…O35	2.86	0.0054	0.018	0.00071	-0.81	0.15	vdW
	17	H180…O34	2.34	0.0114	0.040	0.00131	-0.85	0.14	Weak H-bond
	18	H175…O119	2.96	0.0046	0.016	0.00070	-0.79	0.31	vdW
	19	Cl186…H114	3.22	0.0038	0.012	0.00062	-0.72	0.45	vdW
	20	Cl186…H133	3.81	0.0011	0.003	0.00023	-0.64	0.12	vdW
	21	Cl186…H154	3.47	0.0020	0.007	0.00042	-0.66	0.07	vdW
	22	H179…O119	2.41	0.0109	0.036	0.00106	-0.87	0.02	Weak H-bond
	23	H179…H114	2.40	0.0052	0.016	0.00070	-0.79	0.68	vdW
	24	Cl182…O119	3.40	0.0060	0.022	0.00087	-0.81	0.48	vdW
	25	Cl185…O34	3.54	0.0051	0.016	0.00068	-0.80	0.28	vdW
	26	Cl185…H9	3.01	0.0053	0.017	0.00085	-0.75	0.07	vdW
	27	Cl185…H154	3.76	0.0015	0.004	0.00027	-0.68	0.97	vdW
	28	Cl185…H135	2.71	0.0096	0.031	0.00139	-0.78	0.05	vdW
	29	Cl185…H156	3.26	0.0027	0.009	0.00053	-0.68	0.20	vdW
**β-HCH@C1-γ-CD.2**	1	Cl183…H93	3.00	0.0055	0.017	0.00090	-0.74	0.11	vdW
	2	Cl183…O99	3.36	0.0062	0.021	0.00082	-0.82	0.14	vdW
	3	Cl183…H91	3.08	0.0048	0.015	0.00077	-0.73	0.06	vdW
	4	Cl183…H114	3.04	0.0040	0.014	0.00079	-0.71	0.09	vdW
	5	Cl183…H112	2.87	0.0066	0.022	0.00110	-0.74	0.09	vdW
	6	H176…O78	3.13	0.0026	0.010	0.00054	-0.71	0.94	vdW
	7	H176…H70	2.45	0.0042	0.013	0.00059	-0.77	0.22	vdW
	8	Cl181…O78	3.80	0.0033	0.011	0.00053	-0.75	0.88	vdW
	9	Cl181…H93	3.22	0.0039	0.012	0.00060	-0.75	0.22	vdW
	10	Cl181…H72	3.29	0.0031	0.010	0.00053	-0.72	0.17	vdW
	11	Cl181…H70	2.95	0.0062	0.019	0.00093	-0.76	0.04	vdW
	12	H178…O80	2.31	0.0125	0.044	0.00135	-0.86	0.09	Weak H-bond
	13	H180…H70	2.30	0.0061	0.020	0.00085	-0.79	0.40	vdW
	14	H180…H79	2.35	0.0057	0.020	0.00099	-0.76	0.38	vdW
	15	Cl184…H165	3.12	0.0049	0.015	0.00078	-0.75	0.39	vdW

	16	Cl184…H133	3.35	0.0032	0.009	0.00048	-0.75	0.03	vdW
	17	Cl184…H142	3.39	0.0032	0.011	0.00066	-0.69	1.60	vdW
	18	H177…H154	2.32	0.0048	0.016	0.00079	-0.75	0.14	vdW
	19	Cl186…H154	3.13	0.0056	0.017	0.00079	-0.78	0.52	vdW
	20	Cl186…H163	2.78	0.0086	0.028	0.00128	-0.78	0.14	vdW
	21	Cl186…O162	3.93	0.0027	0.009	0.00047	-0.72	0.59	vdW
	22	Cl186…H18	3.34	0.0028	0.009	0.00050	-0.69	0.12	vdW
	23	H179…O162	3.24	0.0019	0.007	0.00047	-0.66	1.29	vdW
	24	Cl182…O15	3.76	0.0032	0.011	0.00057	-0.74	0.22	vdW
	25	Cl182…H30	2.79	0.0067	0.023	0.00117	-0.75	0.05	vdW
	26	Cl182…H28	2.88	0.0071	0.023	0.00105	-0.77	0.07	vdW
	27	Cl182…O36	3.23	0.0081	0.028	0.00094	-0.85	0.10	vdW
	28	Cl182…H49	2.92	0.0067	0.021	0.00098	-0.77	0.05	vdW
	29	Cl182…H51	2.99	0.0059	0.018	0.00090	-0.76	0.14	vdW
	30	Cl185…H7	2.98	0.0056	0.018	0.00093	-0.74	0.06	vdW
	31	Cl185…H39	3.23	0.0044	0.016	0.00083	-0.73	4.46	vdW
	32	Cl185…H28	2.82	0.0083	0.026	0.00120	-0.78	0.12	vdW
	33	Cl185…H60	3.23	0.0038	0.012	0.00065	-0.73	0.48	vdW
	34	Cl185…H49	3.36	0.0034	0.015	0.00047	-0.76	0.43	vdW
**β-HCH@C4-α-CD**	1	Cl143…H58	2.78	0.0080	0.030	0.00146	-0.76	0.26	vdW
	2	Cl143…H49	2.85	0.0076	0.025	0.00115	-0.78	0.14	vdW
	3	Cl143…H81	2.98	0.0071	0.025	0.00125	-0.75	2.44	vdW
	4	Cl140…H79	3.07	0.0048	0.015	0.00082	-0.72	0.12	vdW
	5	H137…O101	2.37	0.0114	0.041	0.00139	-0.84	0.06	Weak H-bond
	6	H133…O101	2.20	0.0158	0.061	0.00205	-0.84	0.17	Weak H-bond
	7	H135…O101	2.42	0.0109	0.037	0.00112	-0.86	0.17	Weak H-bond
	8	H136…H37	2.30	0.0044	0.014	0.00066	-0.76	-1.56	vdW
	9	Cl144…H28	2.85	0.0077	0.025	0.00115	-0.77	0.15	vdW
	10	Cl144…H91	3.38	0.0028	0.008	0.00047	-0.70	0.06	vdW
	11	Cl144…H39	3.16	0.0053	0.018	0.00092	-0.75	0.44	vdW

	12	Cl144…H7	3.05	0.0049	0.015	0.00083	-0.72	0.10	vdW
	13	Cl144…H112	3.09	0.0046	0.014	0.00078	-0.72	0.08	vdW
	14	Cl142…H7	3.12	0.0054	0.018	0.00084	-0.77	1.99	vdW
	15	Cl142…H18	2.80	0.0088	0.034	0.00162	-0.77	1.44	vdW
	16	Cl142…H121	2.73	0.0097	0.032	0.00136	-0.80	0.05	vdW
	17	Cl141…H121	3.16	0.0046	0.014	0.00069	-0.75	0.34	vdW
**β-HCH@C4-β-CD.1**	1	Cl161…H79	3.90	0.0012	0.004	0.00025	-0.66	2.88	vdW
	2	Cl161…H101	3.21	0.0038	0.014	0.00080	-0.70	1.20	vdW
	3	Cl161…H121	3.18	0.0042	0.014	0.00074	-0.72	0.42	vdW
	4	Cl164…H119	3.21	0.0040	0.012	0.00063	-0.74	0.06	vdW
	5	Cl164…H110	3.33	0.0031	0.010	0.00054	-0.72	0.36	vdW
	6	Cl164…H142	3.28	0.0043	0.016	0.00084	-0.72	0.89	vdW
	7	Cl164…H131	2.84	0.0070	0.023	0.00110	-0.76	0.08	vdW
	8	Cl164…H18	3.13	0.0049	0.015	0.00071	-0.77	0.13	vdW
	9	Cl165…H140	2.87	0.0067	0.022	0.00111	-0.75	0.08	vdW
	10	Cl165…H16	2.95	0.0073	0.025	0.00117	-0.77	0.87	vdW
	11	H157…O38	2.48	0.0090	0.031	0.00105	-0.84	0.04	vdW
	12	H159…H58	2.31	0.0078	0.027	0.00109	-0.80	1.51	vdW
	13	H159…O38	2.47	0.0096	0.031	0.00093	-0.87	0.17	vdW
	14	H155…O38	2.43	0.0096	0.034	0.00109	-0.85	0.11	vdW
	15	Cl165…H16	2.95	0.0073	0.025	0.00117	-0.77	0.87	vdW
	16	Cl165…H140	2.87	0.0067	0.022	0.00111	-0.75	0.08	vdW
	17	Cl164…H18	3.13	0.0049	0.015	0.00071	-0.77	0.13	vdW
	18	Cl164…H131	2.84	0.0070	0.023	0.00110	-0.76	0.08	vdW
	19	Cl164…H142	3.28	0.0043	0.016	0.00084	-0.72	0.89	vdW
	20	Cl164…H110	3.33	0.0031	0.010	0.00054	-0.72	0.36	vdW
	21	Cl164…H119	3.21	0.0040	0.012	0.00063	-0.74	0.06	vdW
**β-HCH@C4-β-CD.2**	1	H154…O13	3.04	0.0030	0.011	0.00058	-0.73	1.20	vdW
	2	Cl162…H133	2.87	0.0075	0.023	0.00110	-0.77	0.01	vdW
	3	Cl162…O118	3.38	0.0058	0.021	0.00083	-0.81	0.03	vdW

	4	Cl162…H112	2.59	0.0100	0.036	0.00162	-0.78	0.08	Weak H-bond
	5	Cl162…H110	2.89	0.0066	0.021	0.00103	-0.76	0.08	vdW
	6	Cl162…O99	3.15	0.0095	0.033	0.00102	-0.86	0.15	vdW
	7	Cl162…H93	2.70	0.0097	0.032	0.00138	-0.79	0.02	vdW
	8	Cl162…H91	3.09	0.0050	0.015	0.00074	-0.76	0.33	vdW
	9	Cl160…O77	3.76	0.0033	0.011	0.00055	-0.74	0.19	vdW
	10	Cl163…H91	3.09	0.0049	0.015	0.00075	-0.75	0.15	vdW
	11	Cl163…H72	3.52	0.0049	0.015	0.00070	-0.78	0.33	vdW
	12	Cl163…O78	3.86	0.0031	0.010	0.00048	-0.75	0.95	vdW
	13	H155…O78	2.53	0.0081	0.027	0.00091	-0.84	0.09	vdW
	14	H155…H72	2.39	0.0049	0.015	0.00070	-0.78	0.33	vdW
	15	H159…O77	2.92	0.0041	0.013	0.00062	-0.77	0.07	vdW
	16	H159…H72	2.54	0.0042	0.014	0.00058	-0.79	0.67	vdW
	17	H159…H82	2.24	0.0046	0.017	0.00086	-0.75	0.08	vdW
	18	H157…H72	1.84	0.0141	0.047	0.00179	-0.82	0.08	Weak H-bond[b]
	19	Cl164…O76	3.74	0.0037	0.012	0.00062	-0.74	0.47	vdW
	20	Cl164…H51	2.98	0.0061	0.019	0.00088	-0.77	0.20	vdW
	21	Cl164…O14	3.52	0.0051	0.019	0.00085	-0.79	0.74	vdW
	22	Cl165…H51	2.69	0.0105	0.034	0.00140	-0.80	0.07	Weak H-bond
	23	Cl165…H49	2.98	0.0062	0.019	0.00089	-0.77	0.15	vdW
	24	Cl165…O36	3.04	0.0110	0.040	0.00122	-0.86	0.18	vdW[a]
	25	Cl165…H30	2.91	0.0066	0.021	0.00098	-0.77	0.20	vdW
	26	Cl165…H28	2.61	0.0099	0.035	0.00158	-0.78	0.02	vdW
	27	Cl165…O15	3.11	0.0104	0.036	0.00101	-0.87	0.23	vdW[a]
	28	Cl165…H20	2.59	0.0106	0.036	0.00151	-0.80	0.03	Weak H-bond
	29	Cl165…H9	2.76	0.0092	0.029	0.00124	-0.79	0.12	vdW
	30	H158…O14	2.69	0.0069	0.023	0.00073	-0.85	0.32	vdW
	31	H158…O13	2.41	0.0106	0.036	0.00106	-0.87	0.08	Weak H-bond
	32	H156…H9	2.33	0.0052	0.016	0.00073	-0.78	0.42	vdW

**β-HCH@C4-γ-CD**	1	Cl186…H125	2.80	0.0074	0.027	0.00121	-0.78	0.23	vdW
	2	H177…O120	2.83	0.0042	0.015	0.00073	-0.75	0.12	vdW
	3	H177…H133	2.42	0.0046	0.015	0.00064	-0.79	0.19	vdW
	4	H175…H114	2.54	0.0030	0.010	0.00059	-0.69	0.10	vdW
	5	H179…O119	2.68	0.0067	0.022	-0.00394	-0.83	0.27	vdW
	6	H179…H114	2.40	0.0050	0.016	0.00076	-0.77	0.18	vdW
	7	H179…H124	2.36	0.0041	0.016	0.00077	-0.76	0.48	vdW
	8	Cl182…O118	3.59	0.0046	0.016	0.00072	-0.77	0.28	vdW
	9	Cl182…H93	2.96	0.0051	0.017	0.00094	-0.71	0.11	vdW
	10	Cl182…O78	3.79	0.0028	0.010	0.00056	-0.71	20.97	vdW
	11	Cl182…H70	2.98	0.0056	0.018	0.00090	-0.74	0.07	vdW
	12	Cl182…O57	3.52	0.0050	0.017	0.00071	-0.80	0.16	vdW
	13	Cl182…H51	2.81	0.0077	0.025	0.00121	-0.76	0.03	vdW
	14	Cl185…H51	3.21	0.0045	0.014	0.00064	-0.78	0.89	vdW
	15	Cl185…O55	3.46	0.0057	0.020	0.00083	-0.79	0.27	vdW
	16	Cl185…H41	2.95	0.0059	0.020	0.00086	-0.79	0.58	vdW
	17	Cl181…H70	3.24	0.0036	0.011	0.00060	-0.73	0.33	vdW
	18	Cl181…H49	3.61	0.0018	0.005	0.00032	-0.67	0.02	vdW
	19	Cl181…H28	4.20	0.0006	0.002	0.00014	-0.57	0.22	vdW
	20	H180…O36	2.88	0.0039	0.013	0.00066	-0.76	0.07	vdW
	21	H180…H30	2.57	0.0033	0.010	0.00056	-0.72	0.54	vdW
	22	H178…H30	2.05	0.0093	0.032	0.00137	-0.80	0.30	vdW
	23	H178…O34	2.54	0.0081	0.030	0.00109	-0.83	0.80	vdW
	24	H176…O15	3.14	0.0022	0.008	0.00052	-0.67	0.17	vdW
	25	Cl184…H9	2.79	0.0080	0.026	0.00125	-0.77	0.10	vdW
	26	Cl184…H7	3.00	0.0060	0.018	0.00085	-0.77	0.05	vdW
	27	Cl184…O162	3.20	0.0079	0.028	0.00097	-0.84	0.08	vdW
	28	Cl184…H156	2.88	0.0060	0.020	0.00103	-0.74	0.09	vdW
	29	Cl184…H154	2.78	0.0083	0.027	0.00126	-0.77	0.04	vdW
	30	Cl184…O141	3.63	0.0042	0.014	0.00064	-0.78	0.12	vdW

	31	Cl183…H7	3.30	0.0033	0.010	0.00055	-0.73	0.67	vdW
	32	Cl183…H18	3.56	0.0022	0.007	0.00041	-0.70	0.71	vdW
	33	Cl183…H154	3.06	0.0052	0.016	0.00078	-0.75	0.25	vdW
	34	Cl183…H133	3.02	0.0059	0.018	0.00082	-0.77	0.15	vdW
	35	Cl183…H142	3.27	0.0042	0.015	0.00085	-0.71	0.73	vdW

a Halogen bond, interaction with the electron density of a H-bond between a halogen atom and a more electronegative atom (chlorine and oxygen in this particular case) that is due to the anisotropy of the electronic density of the halogen.

b Dihydrogen bond, H-bond that is established between two H atoms with a high difference on theirs partial charges.

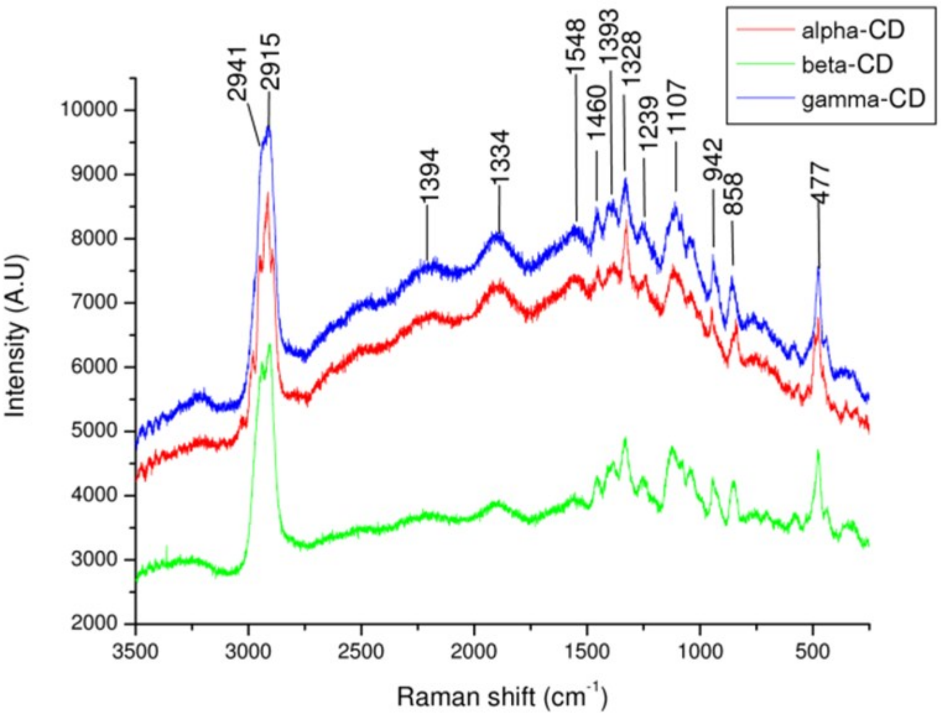

Figure S3. Resonance Raman spectra of the different cyclodextrins α-CD in black, β-CD in red, and γ-CD in green.

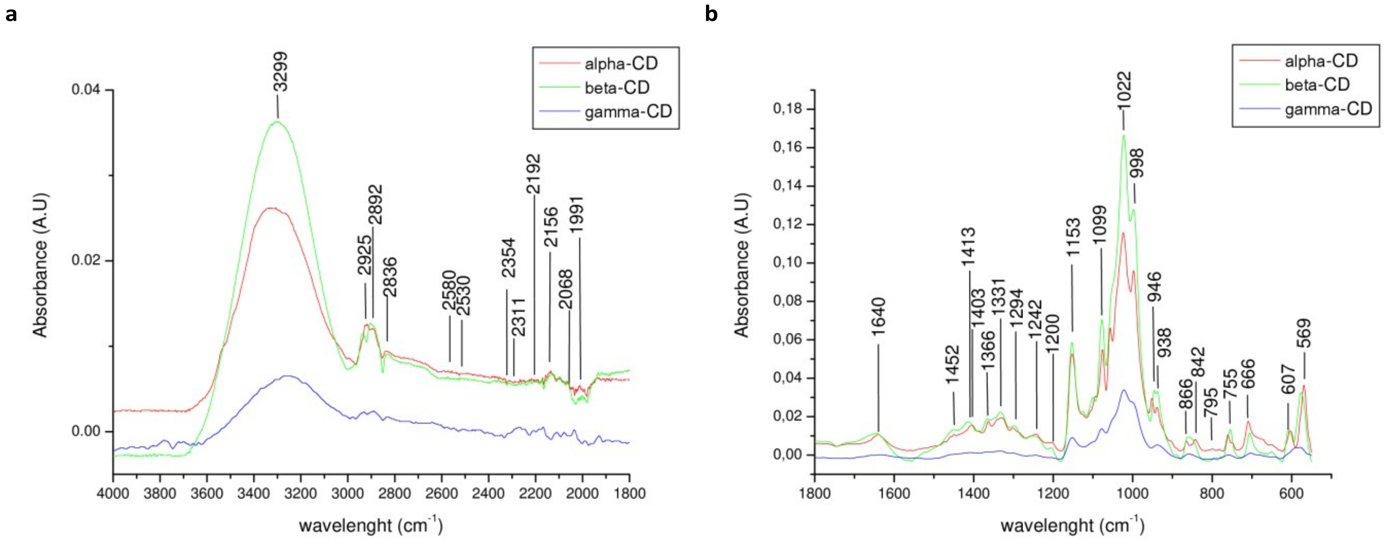

Figure S4. ATR-FTIR spectra of the region of the absorption peak of the CDs. (a) At the left in the region of 4000-1800 cm-1 and (b) at the right in the region 1800-500cm-1 (α in red, β in green, and γ in blue)

Table S8. Assignments of the absorption bands (cm^{-1}) in Raman

β-HCH (cm^{-1})	α-CD (cm^{-1})	β-CD (cm^{-1})	γ-CD (cm^{-1})	β-HCH@α-CD (cm^{-1})	β-HCH@β-CD (cm^{-1})	β-HCH@γ-CD (cm^{-1})	assignments
2938	2941; 2915	2941; 2915	2941; 2915	2938	2938	2938	ν_{as} (C-H) of $-CH_2$
1330, 1285, 1206 and 1004	-	-	-	1330, 1285, 1206 and 1004	1330, 1285, 1206 and 1004	1330, 1285, 1206 and 1004	ν (C-H) stretching
-	-	-	-	1228; 1190	1228; 1190	1228; 1190	ν (C-O-C) stretch
-	942	942	942	-	-	939	ν_{as} (C-O-C) stretch[2]
848	858	858	858	848	848	848	δ (C-H) out of plane bending
741	-	-	-	741	741	741	δ (C-Cl)
298; 259	-	-	-	298; 259	298; 259	298; 259	δ (C-C)

Table S10. Assignments of the absorption bands (cm^{-1}) in ATR-FTIR

β-HCH (cm^{-1})	α-CD (cm^{-1})	β-CD (cm^{-1})	γ-CD (cm^{-1})	β-HCH@α-CD (cm^{-1})	β-HCH@β-CD (cm^{-1})	β-HCH@γ-CD (cm^{-1})	assignments
-	~3300	~3326	~3259	-	-	-	ν (O…H) stretch
2988; 2975; 2964; 2933	2925	2925	2925	2963; 2941	2963; 2941	2963; 2941	ν_{as} (C-H) of -CH2
-	-	-	-	2644-2397	2644-2397	2644-2397	new bands
	1639	1643[3]	-	-	-	-	ν (O-H) bending
1341	-	-	-	-	-	-	ν (C-H) bending, rocking
-	-	-	-	1228; 1190	1228; 1190	1228; 1190	ν (C-O-C) stretch
-	1153; 1078	1153; 1078	1153; 1078	-	-	-	ν_{as} (C-O-C) stretch[4]
778-666	-	-	-	-	-	-	ν (C-Cl)

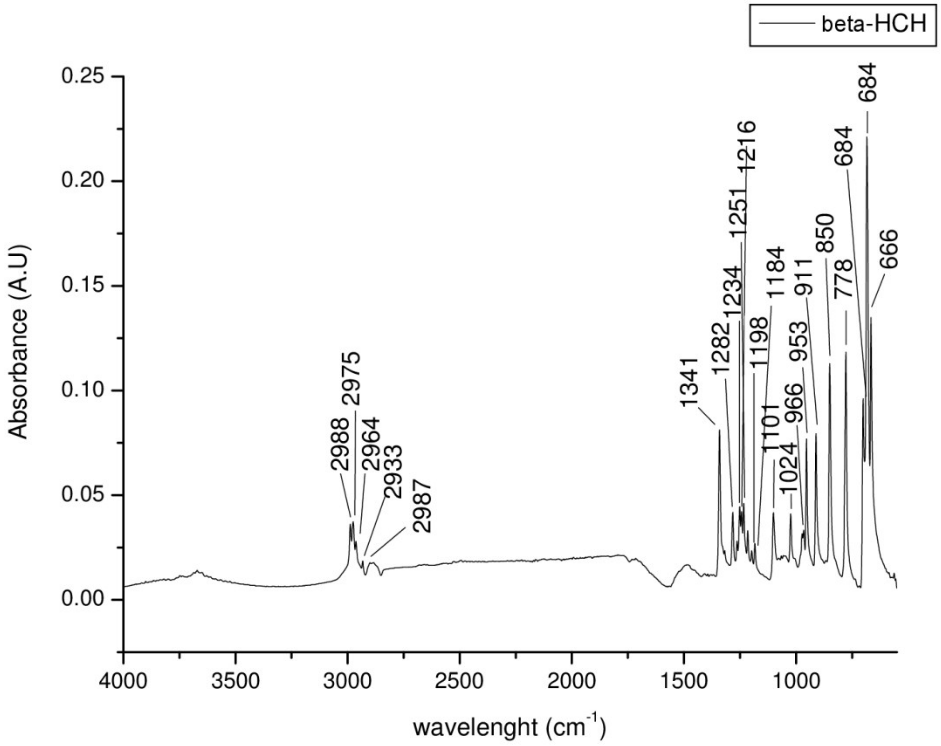

Figure S5. ATR-FTIR spectrum of β-HCH in 4000-550 cm^{-1} region.

References

1. Mura, P., Analytical Techniques for Characterization of Cyclodextrin Complexes in Aqueous Solution: A Review. *J. Pharm. Biomed. Anal.* **2014,** *101*, 238-250.

2. Prosser, A. J. and Franses, E. I., Infrared Reflection Absorption Spectroscopy (Irras) of Aqueous Nonsurfactant Salts, Ionic Surfactants, and Mixed Ionic Surfactants. *Langmuir.* **2002,** *18* (24), 9234-9242.

3. Ghorpade, M. V. S., Preparation and Evaluation of Domperidone/B-Cyclodextrin/Citric Acid/Mannitol Quaternary Inclusion Complex: An in Vitro Study. *Asian J. Pharm.* **2016,** *10* (03).

4. Cannavà, C.; Crupi, V.; Ficarra, P.; Guardo, M.; Majolino, D.; Mazzaglia, A.; Stancanelli, R. and Venuti, V., Physico-Chemical Characterization of an Amphiphilic Cyclodextrin/Genistein Complex. *J. Pharm. Biomed. Anal.* **2010,** *51* (5), 1064-1068.
